# An early response regulatory cluster induced by low temperature and hydrogen peroxide in seedlings of chilling-tolerant japonica rice

**DOI:** 10.1186/1471-2164-8-175

**Published:** 2007-06-18

**Authors:** Chen Cheng, Kil-Young Yun, Habtom W Ressom, Bijayalaxmi Mohanty, Vladimir B Bajic, Yulin Jia, Song Joong Yun, Benildo G de los Reyes

**Affiliations:** 1 Department of Biological Sciences, University of Maine, Orono, ME 04469, USA; 2 Lombardi Comprehensive Cancer Research Center, Georgetown University, Washington, DC 20057, USA; 3 Institute for Infocomm Research, 21 Heng Mui Keng Terrace, 119613, Singapore; 4 South African National Bioinformatics Institute, University of the Western Cape, Bellville, 7535, South Africa; 5 USDA-ARS, Dale Bumpers National Rice Research Center, Stuttgart, AR 72160, USA; 6 Division of Biological Resources Sciences and Institute of Agricultural Science and Technology, Chonbuk National University, Jeonju 561-756, South Korea

## Abstract

**Background:**

Plants respond to low temperature through an intricately coordinated transcriptional network. The *CBF/DREB*-regulated network of genes has been shown to play a prominent role in freeze-tolerance of *Arabidopsis *through the process of cold acclimation (CA). Recent evidence also showed that the *CBF/DREB *regulon is not unique to CA but evolutionarily conserved between chilling-insensitive (temperate) and chilling-sensitive (warm-season) plants. In this study, the wide contrast in chilling sensitivity between indica and japonica rice was used as model to identify other regulatory clusters by integrative analysis of promoter architecture (*ab initio*) and gene expression profiles.

**Results:**

Transcriptome analysis in chilling tolerant japonica rice identified a subset of 121 '*early response*' genes that were upregulated during the initial 24 hours at 10°C. Among this group were four transcription factors including *ROS-bZIP1 *and another larger sub-group with a common feature of having as1/ocs-like elements in their promoters. Cold-induction of *ROS-bZIP1 *preceded the induction of as1/ocs-like element-containing genes and they were also induced by exogenous H_2_O_2 _at ambient temperature. Coordinated expression patterns and similar promoter architectures among the '*early response*' genes suggest that they belong to a potential regulon (*ROS-bZIP – as1/ocs *regulatory module) that responds to elevated levels of ROS during chilling stress. Cultivar-specific expression signatures of the candidate genes indicate a positive correlation between the activity of the putative regulon and genotypic variation in chilling tolerance.

**Conclusion:**

A hypothetical model of an ROS-mediated regulon (*ROS-bZIP – as1/ocs*) triggered by chilling stress was assembled in rice. Based on the current results, it appears that this regulon is independent of ABA and *CBF/DREB*, and that its activation has an important contribution in configuring the rapid responses of rice seedlings to chilling stress.

## Background

Transcriptional regulation is an important aspect of the complex genetic and biochemical networks involved in plant responses to low temperature. In plants like *Arabidopsis *that evolved to withstand freezing by cold acclimation (CA), the transcriptional regulatory network is defined by the interaction between a number of cold-responsive transcription factors and their cognate cis-elements in the promoters of a suite of downstream target genes [1-4]. The downstream target genes, which are activated in concert not only by low temperature but also by other related environmental (dehydration, high salinity) and chemical (abscisic acid or ABA) signals were referred to as *COR *for cold regulated, *rd *for responsive to desiccation, *lti *for low temperature induced or *kin *for cold-inducible [5-8]. Evidence supporting the important roles of *COR *genes in cellular protection against the common biochemical perturbations caused by low temperature and dehydration via protein chaperoning (e.g., LEA proteins), membrane stabilization (e.g., COR15a) and osmotic adjustment (e.g., Δ^1^-pyrroline-5-carboxylate synthase) has been established by transgenic analysis [9-11].

Molecular dissection of the promoters of several *COR *genes of *Arabidopsis *(e.g., *rd29A/lti78/cor78*, *rd17/cor47*, *cor15a*, *cor6.6/kin2*) indicated that they are regulated in an ABA-dependent or ABA-independent pathway under cold, dehydration and high salinity conditions. However, their cold-induced expression is largely through the ABA-independent pathway mediated by highly conserved promoter cis-elements that function as binding sites for transcriptional activators [6,12]. For example, ABA-responsive expression of *rd29A/lti78/cor78 *requires a highly conserved promoter motif PyACGTGGC called ABA-Response Element (ABRE). On the other hand, low temperature, dehydration and high salt- inducible expression of *rd29/lti78/cor78 *requires a 9-bp sequence TA(C/G)CGACAT called C-repeat/Dehydration-Responsive Element (CRT/DRE) that is not independently activated by ABA alone [8]. Many *Arabidopsis *genes with ABRE-like and CRT/DRE-like motifs in their promoters have been identified and these genes have been characterized at least in terms of their stress-responsive expression [10,13].

The transcription factor(s) that interact with the ABRE (AREB or ABF) are bZIP-type proteins that belong to a specific group (Class A) of stress-related bZIP proteins [14,15]. The activators of CRT/DRE-containing genes belong to a plant-specific group of AP2/EREBP-type DNA-binding proteins referred to as *CBF1/DREB1b*, *CBF2/DREB1c*, *CBF3/DREB1a, CBF4, DREB2A *and *DREB2 *[12,16,17]. Overexpression of either *CBF1*/*DREB1b *or *CBF3*/*DREB1a *in *Arabidopsis *resulted in strong and coordinated activation of the target *COR *genes, and subsequently CA-independent tolerance freezing and dehydration [18,19]. Thus, the CBF/DREB regulon was the first example and most prominent regulatory cluster associated with cold stress in plants.

More recently, global gene expression profiling experiments revealed that a large number of *Arabidopsis *genes are responsive to low temperature. Of the more than 500 candidates so far identified, about 60% and 40% were upregulated and downregulated during CA, respectively [13,20]. Analysis of the transcriptome of *CBF*-overexpressing *Arabidopsis *revealed that the *CBF/DREB *regulon is a large cluster comprised of about a hundred *CBF/DREB*-responsive genes that includes both the known *COR *genes as well as many newly identified members. Potential sub-regulons defined by *RAP2.1 *and *RAP2.6 *transcription factors were also identified and presumed to function under the larger *CBF/DREB *regulon [10,13]. Furthermore, transcriptome data also revealed that the low temperature genetic network involves other regulatory clusters in addition to the *CBF/DREB *regulon. An example of this is the *ZAT12 *regulon, which is comprised of more than 20 downstream target genes. Candidate cognate enhancers in the promoters of *ZAT12*-target genes have also been identified. Potential target sites of this transcription factor contain the core motif CATTG [1,20].

Although the precise hierarchical organization of the low temperature genetic networks is yet to be defined, it appears that *CBF/DREB *and *ZAT12 *pathways (and perhaps other unidentified pathways) constitute a '*super regulon*' that configure the overall low temperature stress tolerance mechanisms in *Arabidopsis*. These pathways are further fine-tuned by additional components (e.g., *HOS1, LOS1 *and *LOS2*) either by positive or negative regulation [21,22]. Related studies involving a global survey of metabolic changes also showed that the *CBF/DREB *regulon plays a prominent role in configuring the biochemical status of *Arabidopsis *during exposure to low temperature [11,13,20]. Given the evolutionary conservation of *CBF/DREB *regulon between temperate and warm-season plants, all these findings established the prominent role of this pathway in the cold stress global regulatory networks of plants in general.

Many cold-responsive genes containing CRT/DRE-like promoter elements have also been identified in plants that do not acclimate to freezing, indicating that various cold stress response regulatory modules are highly conserved even between chilling-insensitive (temperate) and chilling-sensitive (warm-season) plants [23-26]. However, despite the current models of the *Arabidopsis *CA regulatory networks and the identification of functional homologs of *CBF/DREB *transcription factors and their corresponding regulatory modules in rice and other warm-season plants, the precise composition and hierarchical organization of the component pathways of the global networks in this group of plants is yet to be defined [27,28].

Indica and japonica rice cultivars exhibit wide contrast in terms of their sensitivity to chilling. At the seedling stage, most japonica cultivars survive continuous exposure to as low as 10°C for up to 7 days better than most indica cultivars. Having a complete genome sequence and a variety of expression profiling platforms, the japonica rice is an ideal model to assemble the various component pathways of the cold stress regulatory networks of chilling-sensitive plants. Parallel assembly of the homologous networks involved in regulating the transcriptome in chilling-insensitive (*Arabidopsis*) and chilling-sensitive (rice) plant models will contribute to the understanding of the evolutionary changes that led to such differences in low temperature sensitivity between the two groups of plants. To contribute to this goal, we performed a semi-global survey of the chilling stress transcriptome of rice using a cDNA microarray representing about 6,000 seedling-expressed genes whose ESTs were enriched in a cold stress subtracted library [29]. The goal of this initial survey was to identify the early components of the network through the identification of a set of co-regulated genes during the initial exposure to 10°C.

The results of the survey presented here revealed an early response regulatory cluster that is potentially independent of the *CBF/DREB *regulon perhaps activated via reactive oxygen species (ROS). Although many ROS inducible genes have been identified in *Arabidopsis *[30], our knowledge of how these genes are organized into regulatory clusters lags behind our understanding of the hierarchical organization of the *CBF/DREB *and other known regulons. Here we present evidence of an ROS-mediated regulatory module that functions as an early component of the chilling stress response pathway in japonica rice based on integrative genomics analysis.

## Results

### Snapshot of the cold stress early response transcriptome of rice

Our previous studies showed that the cold stress library used to assemble the 5,855-feature cDNA microarray was enriched with candidate chilling-upregulated genes [23,29]. Analysis of transcript profiles at 0.5, 2, 6, 12, and 24 hours after the initiation of chilling identified 121 unique features (~2% of the total) that were significantly upregulated in one or more time points at a cut-off level of 1.8-fold. We refer to this subset of candidates as '*early response*' genes (see Additional files 1 and 2). The cut-off level was based on the assumption that induction of early response regulatory genes (transcription factors) occurs at low to moderate levels. A few representatives of the candidate genes identified from this survey were confirmed by northern blot analysis (data not shown).

**Figure 1:**
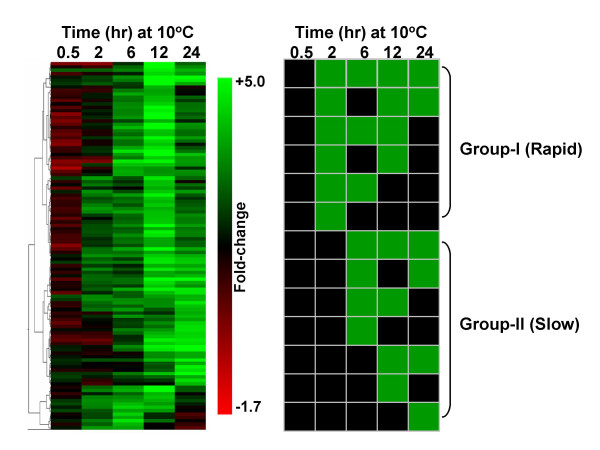
**Summary of microarray analysis showing the expression patterns of chilling stress *'early response' *genes**. Temporal profiles of genes with ≥ 1.8-fold induction in one or more time points during the initial 24 hours at 10°C are shown. Expression values were normalized so thatmean of the ratio of median of all of the features is equal to 1. Left panel – Hierarchical clustering of 121 *'early response' *genes. Temporal expression clusters indicate multiple waves of gene induction. Scale of fold-change values is shown on the right side of the panel. Right panel – Binary hierarchical clustering of '*early response*' genes. The genes induced at or above the 1.8-fold cut-off level were given a value of 1 (green), and those below the cut-off level a value of 0 (black). Clusters indicate that the genes can be categorized into two major groups: *'rapid response' *(Group-I, induction initiated during the initial 2 hours) and *'slow response' *(Group-II, induction initiated after 2 hours). Genes within each group exhibit either a sustained or transient induction during the initial 24 hours under chilling stress (see Additional files 1 and 2 for the list of genes in each group).

The expression profiles of the '*early response*' genes were grouped according to temporal patterns. Hierarchical clustering dendogram shows a complex pattern of gene induction characterized by multiple waves that were detectable as early as 2 hours after the initiation of chilling (Figure 1, left panel). In this scheme, many clusters were established but a meaningful trend was not apparent due to the small number of member genes in each group. We further reduced the branch-complexity of the hierarchical clustering into fewer groups with larger number of member genes by transforming the fold-change values to a binary data (Figure 1, right panel). In this scheme, genes with fold-change values at or above the 1.8-fold cut-off were assigned a value of '1' (green squares) and those below the cut-off a value of '0' (black squares). This clustering scheme showed that the early response upregulated genes can be classified into a '*rapid response*' (Group-I) or '*slow response*' (Group-II) with either sustained or transient induction. Cold-induced expression of Group-I genes started during the initial 2 hours, while induction of Group-II genes did not start until after 2 hours. Of the 121 early response genes, 20 and 101 belong to Group-I and Group-II, respectively (see Additional file 1).

**Figure 2:**
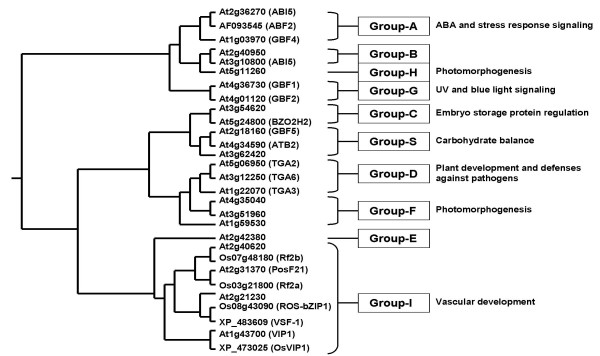
**Phylogenetic tree showing the relationship of rice *ROS-bZIP1 *(Os08g43090) to known classes of plant bZIP transcription factors**. Scheme is based on genome-wide survey and classification of *Arabidopsis *bZIP proteins [15]. *ROS-bZIP1 *belongs to Class-I, which also includes the *RF2a, RF2b *and *VSF-1 *of rice and *VIP1 *of rice and *Arabidopsis*. All *Arabidopsis *genes included in this neighbor-joining tree are identified by standard genomic locus numbers. Phylogenetic tree was assembled using clustalW.

Since our primary goal was to decipher possible '*regulator-target*' gene pairs (regulatory module) that comprise the '*early response*' regulon, we first scrutinized the subset of upregulated genes by searching for candidate transcription factor(s). Of the eight transcription-related genes in this subset, four appeared to be *bona fide *DNA-binding proteins based on characteristic signature domains. Two of the '*early response*' transcription factors (bHLH-type protein – Os01g70310 and bZIP-type protein – Os08g43090) were rapidly induced during the initial 2 hours of chilling (Group-I). The bZIP-type protein, which we refer to as *ROS-bZIP1 *belongs to the Class-I bZIP that includes the *RF2a *and *RF2b *genes of rice. *ROS-bZIP1 *has not been reported previously to be induced by any type of abiotic stress and it is structurally distinct from the other known members of the family of stress-related bZIP transcription factors (Class-A and Class-D) that also include the TGA-type (Figure 2) [15]. The other two '*early response*' transcription factors were *OsMyb4 *(Os04g43680) and an EREBP1-type protein (Os02g54160) distinct from any member of the *CBF/DREB *family. These genes were induced later (around 6 hours after the initiation of chilling treatment) than *ROS-bZIP1 *(Os08g43090) and the bHLH-type protein (Os01g70310), hence classified under Group-II. Except *ROS-bZIP1*, all of these transcription factors have been reported previously to be involved in cold stress either in rice or *Arabidopsis *based on published data or EST and genomic annotations [23,31,32].

In addition to the transcription-related genes that comprised about 7% of the total number of '*early response*' genes identified in this survey, other functional categories were represented as follows: protein synthesis or turnover (21%), intracellular transport and membrane trafficking (13%), metabolism of simple and complex carbohydrates (13%), unknown proteins (11%), stress-related proteins including disease-related (11%), signal transduction (7%), growth and development (6%), metabolism of nitrogenous compounds (4.0%), metabolism of lipids (3.2%) and others including chromatin-related and cellular energetics (4%). These results suggest that rice seedlings respond rapidly to chilling by modulating cellular processes related to growth in order to accommodate the necessary adjustments to the various physiological strains under sub-optimal temperature condition.

### Common regulatory features of '*early response*' genes

The distribution of signature motifs of various cis-elements in the promoters of genes with similar expression profiles often provides corroborating evidence that such group of genes may in fact be co-regulated by the same or related groups of transcription factors. With this reasoning, we surveyed the promoter sequences of the '*early response*' genes with the aim of evaluating the relationships among them in terms of the presence of common sequence motifs with possible function as transcription factor binding sites. The genomic locus of each candidate gene in the microarray was identified by aligning the EST with the Nipponbare reference sequence. BlastN analysis identified 117 genomic loci with high level of confidence (e-value of at least 10^-40 ^or in most cases 0).

Initial search for conserved sequence motifs through the Dragon Motif Builder [33] detected a total of 280 candidates. Motifs obtained in a single run were considered to be highly relevant to this subset of the chilling stress transcriptome if they were present in at least 50% of the promoters. Under this premise, a total of 140 motifs were identified and their potential biological significance inferred by their presence in public promoter databases [34-36]. The results of these analyses revealed at least sixteen motif families to be most likely associated with the regulation of the '*early response*' genes (Table 1). The enrichment levels of these motifs were indicated by the e-value and their percentage occurrence in the target promoters relative to the background sequence. These parameters indicate sufficient enrichment of the candidate motifs in the promoters of the target gene group. The most significant motifs also matched the core sequences of known transcription factors associated with abiotic stress based on established experimental evidence [34-36]. For instance, as we expected the occurrence of the CRT/DRE-like ((C/G)CGAC) core motifs was statistically significant. A ferritin gene (Os11g01530) that has been previously reported as a putative *CBF/DREB *target was among the '*early response*' genes that we found to contain the CRT/DRE [24]. This result was consistent with the expectation based on previous findings that the *CBF/DREB *regulon is activated within the same time period used in the current experiments [23]. Overall, the result of this analysis was nearly identical to the results generated by MEME/MAST and with the motifs detected by manual inspection of representative genes [37]. We concluded that the consensus motifs identified by the search algorithm(s) are likely components of the regulatory modules of the early response transcriptome.

**Table 1:** List of conserved sequence motifs that are enriched in the promoters (-1,000, -1 region) of 117 early response genes.

**Core Motif**	**Transcription Factor**	**Database Annotation**	**% Occurrence**	TIC*	**E-value**
TTTC	GAMyb	Pyrimidine box (barley)	87	10.00	9e-0004
TGACG	bZIP, TGA-type	as1/ocs element (tobacco, Arabidopsis) Auxin response element (soybean)	84	8.29	4e-003
GATGA	bZIP, TGA-type	Xenobiotic stress element (tobacco)	84	9.07	2e-003
TTGATC	WRKY bZIP, TGA-type	W-box (Arabidopsis) as1/ocs element (Arabidopsis) TGA1a factor (tobacco)	79	10.24	9e-004
TCCCAT	ARD	Auxin-response element (soybean)	77	8.88	4e-004
CAAACC	Myb, GARE-type	GA response element (barley) ABRE (Brassica)	72	10.43	8e-004
CAACAA	Myb2	MybR (rice, barley)	71	9.50	1e-003
CACGTG	bZIP	G-box (soybean, tobacco) ABRE (maize, barley)	71	8.66	8e-004
CAACCT	Myc/AP2	MycR (rice)	70	9.59	2e-003
ATCCGG	Myb, GARE-type	GA-response element (barley)	69	8.91	3e-003
CTCGC	bZIP	ABRE (rice)	67	9.20	2e-003
GCGCCGC	AP2/EREBP	GCC-box (tobacco)	66	12.80	2e-004
AACCAA	AtMyb2	Dehydration element (Arabidopsis)	63	10.86	9e-004
GCCGCCG	AP2/EREBP	GCC box (Arabidopsis) ABA response element (maize) Ethylene response element (Arabidopsis)	61	12.64	7e-004
GCGAC	AP2/EREBP, CBF/DREB	CRT/DRE-like (rice, Arabidopsis)	61	9.24	2e-003
ACAAAAT	GAMyb	GAMyb (rice, barley)	46	11.93	4e-004

*Total Information Content

In addition to *CBF/DREB *modules, two other prominent regulatory features of the '*early response*' genes are highlighted in this report (Tables 1 and 2). First, different types of Myb-target motifs (MybR) occur at relatively high frequencies. This includes the pyrimidine box, GARE, CAAC-box, Myb2-box and GAMyb. The second highly conserved feature is the prominence of TGACG, GATGA and TTGATC motifs, many of which are located within the -700 bp regions. TGACG is the signature sequence of the as1/ocs element that functions as binding site of a TGA-type bZIP transcription factor under various conditions that induce oxidative stress. Similarly, the GATGA motif is another known target of a TGA-type bZIP known to respond to xenobiotic stress, which also involves reactive oxygen species (ROS). TTGATC is the signature sequence of W-box found in the promoters of WRKY-target genes, which are activated by pathogen attack and oxidative burst. This promoter element has also shown to be regulated by the same group of TGA-type bZIPs that bind to the as1/ocs elements (as1/ocs-like) [38,39].

**Table 2:** Promoter motifs identified by *ab initio *method using the Dragon Motif Builder program with EM1 and EM2 option

**Sequence Motif**	**Location**	**EM1-threshold**	**EM2-threshold**
TTTTC	Upstream (-1000, -1)	0.875	
TGACG	Upstream (-1000, -1)	0.875	
GATGA	Upstream (-1000, -1)	0.875	
TTGATC	Upstream, downstream (-1000, +350)	0.850	
TCCCAT	Upstream (-500, -1)		0.850
CAAACC	Upstream, downstream (-500, +350)	0.850	
CAACAA	Upstream (-500, -1)		0.850
CACGTG	Upstream (-500, -1)		0.850
CAACCT	Upstream and downstream	0.850	
ATCCGG	Upstream (-1000, -1)	0.875	
CTCGC	Upstream (-1000, -1)	0.875	
GCGCCGC	Upstream, downstream (-1000, +350)	0.875	
AACCAA	Upstream (-1000, -1)		0.850
GCCGCCG	Upstream, downstream (-500, +200)		0.850
GCGAC	Upstream, downstream (-500, +200)	0.875	
ACAAAAT	Upstream (-1000, -1)	0.875	

### Assembly of ROS-mediated regulatory module

Enrichment of the signature sequences of certain families of regulatory elements (including as1/ocs-like motifs) in the promoters of the '*early response*' group of genes (Tables 1 and 2) established a rationale that these genes are possible components of a certain regulatory cluster of the early response transcriptome. As a first step in testing this hypothesis, we performed detailed examinations of a representative subset of Group-I and Group-II genes in order to determine how they may be associated to each other in terms of possible '*cause and effect*' type of relationships. Differences in the temporal sequence and magnitude of gene induction were compared within each group by quantitative PCR (qPCR), and similarities and differences in expression patterns were correlated to the distribution and enrichment of putative promoter cis-elements. The general premise is that the expression of a given transcription factor precedes the induction of its downstream regulatory cluster, and that such cluster contains a consensus element as binding site for the common transcriptional activator.

We have also shown in our previous report that the cultivars CT6748-8-CA-17 (japonica) and INIAP12 (indica) exhibit a wide contrast in chilling sensitivity with the former being more tolerant than the latter [23]. We reasoned that comparing the activity of the putative regulatory cluster between these two cultivars should provide further justification for the potential significance of the predicted regulatory cluster to genotypic variation in chilling tolerance. We examined all four transcription factors (*ROS-bZIP1 *– Os08g43090; bHLH-type protein – Os01g70310; EREBP1-type protein – Os02g54160; *OsMyb4 *– Os04g43680) and five other non-transcription factors (drought-induced high mobility group (HMG) protein – Os06g51220; ferritin – Os11g01530; putative ATP sulfurylase – Os03g53230; an expressed protein similar to ATP synthase – Os12g07140; auxin-induced protein – Os01g13030) that we identified in the microarray survey (see Additional file 1). These genes were specifically chosen for further analysis because they have been previously validated by northern blot (data not shown).

**Figure 3:**
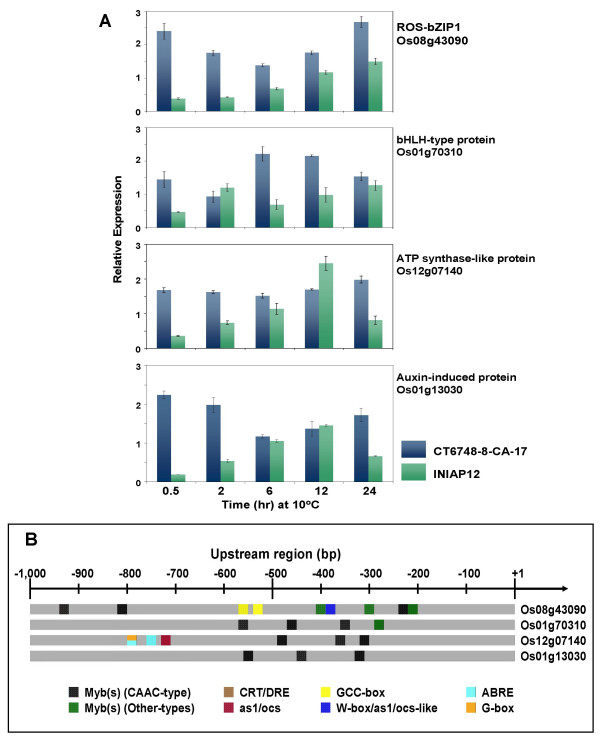
**Analysis of transcription factors (*ROS-bZIP1*, bHLH-type protein) and other non-regulatory genes (ATP synthase-like protein, auxin-induced protein) that belong to the '*rapid early response *' group (Group-I)**. **(A) **Temporal expression profiles by qPCR. Transcript levels at each time point were normalized against the expression of constitutively expressed actin gene. Relative expression of control (0 hour; not shown in the graph) was set to zero by subtracting the values from the relative expression values of each treatment time points. Relative expression values are averages of independent replicates (n = 3; ± SE). The cultivar- specific expression signatures of this group was positively correlated with genotypic variation in chilling tolerance (tolerant – CT6748-8-CA-17; intolerant – INIAP12). Early induction of the ATP synthase-like protein and auxin-induced protein was detected by qPCR but not by microarray. **(B) **Promoter maps of representative Group-I genes showing the relative position and distribution of known stress-responsive cis-element motifs within the -1,000 bp region. Putative MybR sequences are the prominent group of motifs within the -700 bp region.

The temporal grouping of the representative subset of '*early response*' genes according to the qPCR data was generally consistent with the initial grouping based on microarray profile except for the ATP synthase-like protein (Os12g07140) and auxin-induced protein (Os01g13030) whose induction within the initial 2 hours was evident only in the qPCR data. Based on these results, the composition of Group-I was redefined to include these two genes in addition to *ROS-bZIP1 *and the bHLH-type transcription factors (Figure 3A). In CT6748-8-CA-17, all four genes were activated early as shown by their induction peaks within the initial 2 hours. Transcript levels remained significantly higher than basal (control) levels during the entire 24 hours of exposure to chilling. *ROS-bZIP1 *also had a second induction peak that occurred at the end of the 24 hours exposure to chilling. In contrast, expression levels of the representative Group-I genes in INIAP12 were generally several fold lower, less robust, and delayed compared to CT6748-8-CA-17. These patterns were reproducible in multiple tests with independent replicates. Analysis of the promoter structures (-1,000 bp region) of this representative subset of Group-I genes based on the Nipponbare reference sequence showed a prominence of MybR motifs (different types combined) within the -600 bp region (Figure 3B). Chilling tolerance of Nipponbare is comparable to CT6748-8-CA-17 and its expression signatures for the Group-I genes including *ROS-bZIP1 *and bHLH-type transcription factors were also very similar to CT6748-8-CA-17 (data not shown). The as1/ocs-like sequences also occurred in this group of genes but the enrichment level was not as significant as the MybR-related sequences.

**Figure 4:**
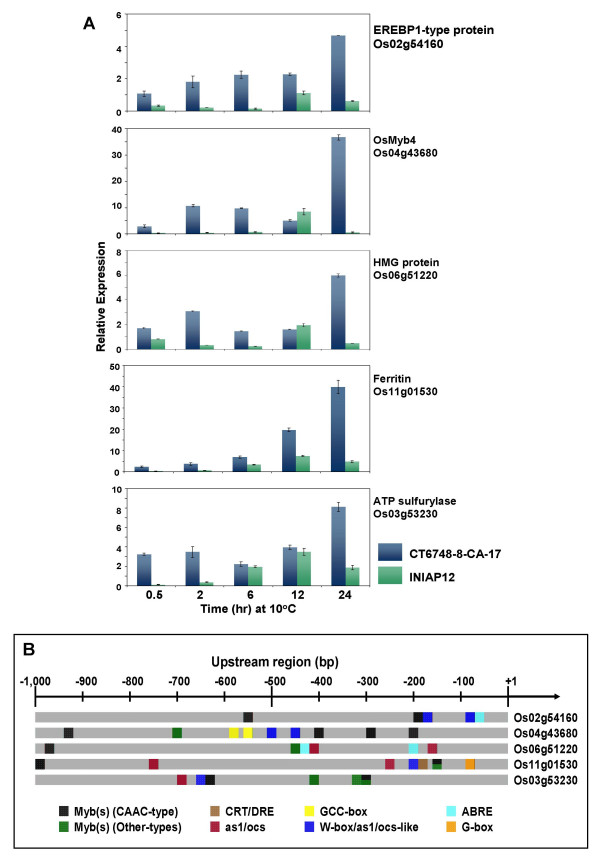
**Analysis of representative '*slow early response*' genes (Group-II) including *OsMyb4*, EREBP1-type protein, high mobility group (HMG) protein, ferritin, and putative ATP sulfurylase. (A) **Expression profiles based on qPCR showing the induction peak(s) of the Group-II genes towards the end of the 24 hours incubation at 10°C. Expression signature of the tolerant (CT6748-8-CA-17) and intolerant (INIAP12) cultivars are shown. Transcript level at each time point was normalized against the expression of constitutively expressed actin gene. Relative expression of control (0 hour; not shown in the graph) was set to zero by subtracting from the relative expression values of each time points. Relative expression values are averages of independent replicates (n = 3; ± SE). **(B) **Promoter maps of representative Group-II genes showing the relative position and distribution of known stress-responsive cis-element motifs. The as1/ocs-like motifs are the prominent family of elements in this group of genes.

The qPCR expression profiles of the representative subset of Group-II genes that include two transcription factors (*OsMyb4 *and an EREBP1-type protein) and three non-transcription factors (HMG protein, ferritin, and putative ATP sulfurylase) confirmed that their cold induction occurred at much slower pace than the Group-I genes. Nevertheless, the differential expression patterns of Group-II genes between CT6748-8-CA-17 and INIAP12 were very similar to the cultivar patterns observed on the Group-I genes (Figure 4A). For instance, CT6748-8-CA-17 exhibited steady increases in the expression of all five genes from the onset of chilling and reached the highest level at 24 hours. In contrast, activity of the Group-II genes in INIAP12 was several fold lower compared to CT6748-8-CA-17 at all time points, thus cold induction in this cultivar was much weaker and in some cases appeared transient. These patterns were quite reproducible in multiple tests with independent replicates.

Apart from the fact that the representative subset of Group-II genes exhibit similar temporal induction patterns, *ab initio *analysis of their promoters based on the Nipponbare reference sequence showed another common feature, defined by the presence of two closely related signature sequences characteristic of the as1/ocs element and W-box/as1/ocs-like element. Either or both of these signature sequences were found at least within the -800 bp region of this representative subset of Group-II genes (Figure 4B, Table 1). Among the motif families detected on the Group-II promoters, which include CRT/DRE, G-box, GCC-box, ABRE and various types of MybR, the as1/ocs-like motifs were the most prominent (consensus). This enrichment is suggestive of a role of these putative elements in the regulation of these genes.

**Figure 5:**
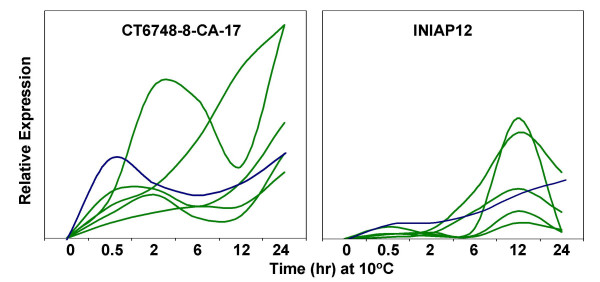
**Composite time-course model illustrating the sequential induction of *ROS-bZIP1 *(blue) and putative downstream target Group-II genes (green) based on the actual profiles shown in Figures. 3A and 4A**. *ROS-bZIP1 *expression preceded the induction of as1/ocs-like element-containing Group-II genes as shown by the very pronounced induction peak within the initial 2 hours in CT6748-8-CA-17 (chilling-tolerant). Induction of the regulatory cluster in INIAP12 (chilling-intolerant) was generally weak and delayed.

Because TGA-type bZIP transcription factors are known to bind to as1/ocs-like motifs [38,40], we hypothesized that between the two transcription factors (*ROS-bZIP1 *and bHLH-type) that were rapidly induced and differentially expressed between CT6748-8-CA-17 and INIAP12, *ROS-bZIP1 *was the more likely regulator of the as1/ocs-like element-containing Group-II genes. We tested this possibility by examining the patterns of sequential induction of *ROS-bZIP1 *and representative subset of as1/ocs-like element-containing Group-II genes by integrating the qPCR expression profiles shown in Figures. 3A and 4A in a composite time-course model (Figure 5). This model showed that *ROS-bZIP1 *expression preceded the induction of Group-II genes as shown by a major induction peak in CT6748-8-CA-17 within the initial 2 hours. The induction peaks of the Group-II genes were evident only after approximately 6 hours and most of the genes reached their highest expression levels at the end of the 24 hours period. This trend suggests that such induction was a likely consequence of the expression of a transcription factor at least during the initial 6 hours. These sequential patterns of induction appeared to occur proportionately with respect to the expression differences observed between CT6748-8-CA-17 and INIAP12.

Based on the relationships suggested by the combined analysis of induction profiles and promoter motif enrichment, it appears that *ROS-bZIP1 *and the as1/ocs-like element-containing Group-II genes are likely components of a possible regulatory cluster (*ROS-bZIP *– as1/ocs regulatory module). Because as1/ocs-like elements are known to play important roles in ROS-responsive gene expression [30,38,41], we reasoned that the functionality of the predicted regulatory module in relation to a possible ROS-mediated signaling can be further justified by demonstrating that the different components of the module can indeed be induced by exogenous ROS. With this reasoning, we studied the effect of exogenous sub-lethal levels of H_2_O_2 _in the absence of cold stress on the expression of the putative *ROS-bZIP *– as1/ocs regulatory module.

**Figure 6:**
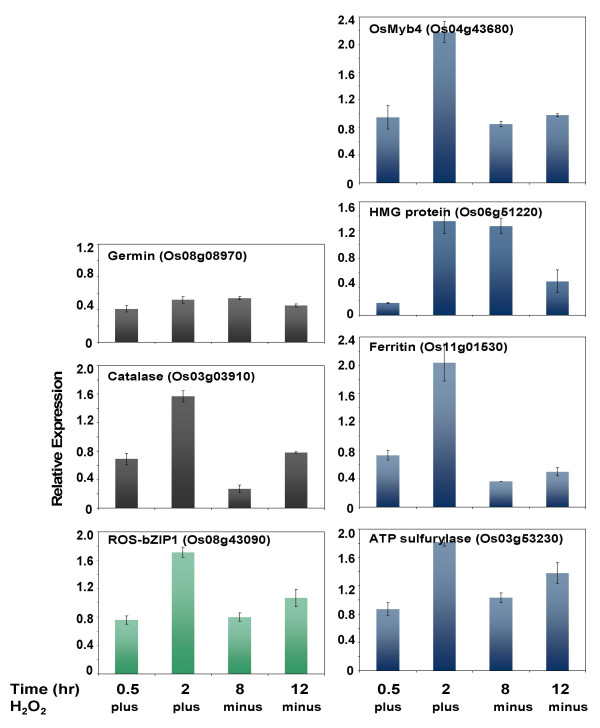
**Effect of exogenous H_2_O_2 _on the expression of the putative ROS-mediated regulatory module that includes *ROS-bZIP1 *and representative genes that contain as1/ocs-like elements in their promoters (*OsMyb4*, high mobility group (HMG) protein, ferritin, and ATP sulfurylase)**. Rice seedlings (CT6748-8-CA-17) were subjected to a continuous 2 hours treatment with 1 mM H_2_O_2_. Transcript levels of H_2_O_2_-treated and non-treated (control) seedlings were monitored at specific time intervals during the 2 hours continuous H_2_O_2 _treatment and another 10 hours after the removal of the exogenous H_2_O_2_. Known H_2_O_2_-responsive gene that contains as1/ocs element-like motifs in its promoter (catalase) and a non-H_2_O_2_-responsive gene that do not contain as1/ocs-like motifs in its promoter (germin) were used as positive and negative controls, respectively. Transcript levels at each time point were normalized against the expression of constitutively expressed actin gene. Relative expression of control (0 hour; not shown in the graph) was set to zero by subtracting from the relative expression values of each treatment time points. Relative expression values are averages of independent replicates (n = 3; ± SE).

Results of the quantitative analysis of gene induction by exogenous H_2_O_2 _are summarized in Figure 6. The expression profile of a known H_2_O_2_-responsive catalase gene (Os03g03910) that contains as1/ocs-elements in its promoter showed that exogenous H_2_O_2 _at a concentration of 1 mM was sufficient to cause a detectable increase in transcript abundance from the basal level within just 2 hours of treatment. This catalase gene was also induced by chilling based on 'unreplicated' microarray data and northern blot (data not shown). In a parallel analysis of a negative control, the expression of a gene that does not contain as1/ocs-like element in its promoter (germin – Os08g08970) in the presence of exogenous H_2_O_2 _was not significantly different from the basal expression level in the absence of exogenous H_2_O_2_.

Of the two Group-I transcription factors, only *ROS-bZIP1 *showed significant H_2_O_2_-induced increase in transcript levels (Figure 6), while exogenous H_2_O_2 _had negligible effects on the expression of the bHLH-type transcription factor (data not shown). The expression profile of *ROS-bZIP1 *was characterized by a steady increase in transcript abundance during the 2 hours of treatment with 1 mM H_2_O_2 _and an immediate drop upon the removal of exogenous H_2_O_2_. Under the same experimental condition, all of the as1/ocs-like element-containing Group-II genes tested (*OsMyb4*, HMG protein, ferritin, and putative ATP sulfurylase) exhibited expression profiles similar to that of *ROS-bZIP1*. The positive control catalase also showed the same response to the removal of exogenous H_2_O_2_, which confirmed that the observed induction in the expression of the candidate ROS-regulated genes are indeed direct effects of exogenous H_2_O_2 _(Figure 6).

The results of the H_2_O_2_-induction experiment were consistent with the hypothesis that the representative '*early response*' genes represent a potential regulatory cluster that responds to both low temperature and ROS (H_2_O_2 _in particular). Given the degree of enrichment of the as1/ocs-like motifs among the 121 candidate genes, this relationship is probably applicable to any member of Group-II genes with similar promoter architecture. These interpretations are of course subject to further confirmation with more direct experimental evidence involving microarray analysis of H_2_O_2_-treated and untreated rice seedlings and direct protein-DNA binding studies.

**Figure 7:**
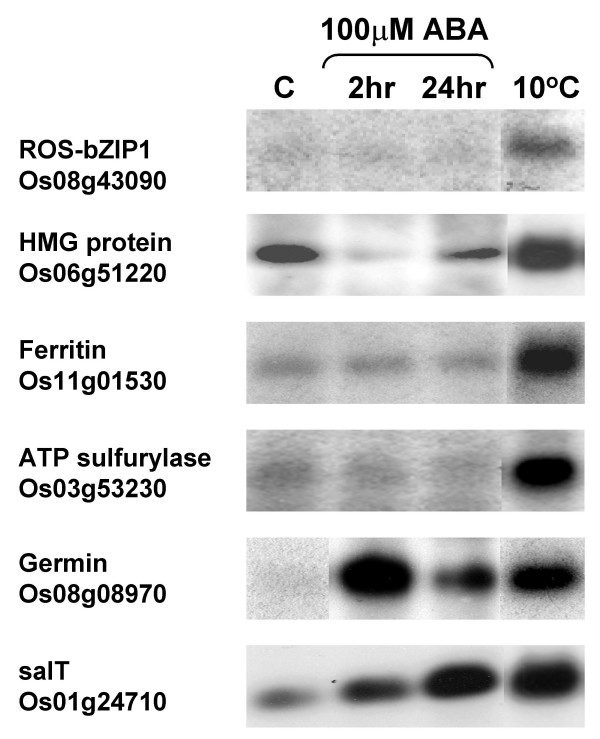
**RNA gel blot analysis showing the effect of exogenous ABA (100 μM) on the expression of representative members of the putative *ROS-bZIP – as1/ocs *regulatory module (*ROS-bZIP1*, high mobility group (HMG) protein, ferritin, and putative ATP sulfurylase)**. Expression of known ABA-inducible genes (germin, salT) was used as positive control for the ABA treatment.

We established based on the integrative analysis of genomics data that the '*early response*' transcriptome associated with chilling stress (in rice at least) involves ROS-mediated gene expression. Abscisic acid (ABA) is another key signal in abiotic stress response signaling in plants and its associated pathway has known linkages with other regulatory groups like the *CBF/DREB *regulon [8]. To further explore whether other chemical signals in addition to ROS (H_2_O_2_) were also involved in the activation of the putative *ROS-bZIP – as1/ocs *regulatory module, we examined the effect of exogenous ABA on the expression of representative subset of H_2_O_2_-inducible genes by northern blot. Results of this test showed that *ROS-bZIP1 *and representative as1/ocs element-containing Group-II genes (HMG protein, ferritin, and putative ATP sulfurylase) were not induced by 100 μM ABA (Figure 7). This concentration of ABA has been shown to induce the expression of cold-responsive rice genes in our previous report [23]. Known ABA-responsive genes (germin – Os08g08970, salT – Os01g24710) showed significant increases in expression under the same concentration and length of exposure to exogenous ABA.

## Discussion

Chilling-induced physiological imbalance leads to elevated levels of reactive oxygen species (ROS) in plant cells [42-45]. In particular, H_2_O_2 _is generated rapidly under stress conditions either by enzymatic means (plasma membrane NADPH oxidase, cell wall peroxidase, amine oxidase) or by the normal metabolic routes in the chloroplast (Mehler reaction) and mitochondria (electron transport and photorespiration). Steady state levels depend on the balance between synthesis and degradation, which is facilitated by the ROS-scavenging system of the cell [44]. With a subset of experimentally validated chilling- and H_2_O_2_-responsive genes, we inferred possible regulatory relationships among a group of genes that were induced in coordinated manner within a short time window during the initial stages of exposure of rice seedlings to 10°C. We hypothesized that the early response transcriptome might involve reactive oxygen species (ROS) via ROS-mediated gene expression defined by the as1/ocs-like promoter elements. Microarray data supports an elevated ROS level in rice seedlings during such period by virtue of the upregulation of ROS scavengers such as catalase, ascorbate peroxidase, glutathione S-transferase, and superoxide dismutase. Transcript levels of these genes were significantly higher than the control levels based on the combined results of microarray, northern blot and RT-PCR analyses (data not shown). Additionally, our current data indicated that a mechanism for effective reduction of the rate of the Fenton reaction is activated during the initial 24 hours based on chilling- and H_2_O_2_-induced expression of a ferritin gene (Os11g01530). The Fenton reaction is responsible for the conversion of H_2_O_2 _to more potent hydroxyl radicals via a Fe^2+^-requiring chemical reaction. Thus, the functional significance of the observed induction of ferritin expression can be explained in terms of its function in Fe^2+ ^sequestration, which would then limit the rate by which unsuccessfully scavenged H_2_O_2 _(which could occur during periods of oxidative burst) would be converted to hydroxyl radicals [46].

It has been established that H_2_O_2 _plays a very important role in mediating signal transduction in response to both abiotic and biotic stresses in plant cells. H_2_O_2 _diffuses rapidly from its site of synthesis within subcellular microdomains depending on concentration and it can transmit intracellular signals to the nucleus by oxidizing various upstream components of the signaling pathway including protein kinases (MAPK cascades), protein phosphatases, transcription factors and membrane-bound calcium-channels. The end response is change in gene expression [47-49]. Although many ROS-inducible genes have been identified in *Arabidopsis *[30], direct involvement of an ROS-mediated regulatory module in the low temperature transcriptional networks has not been studied to the same level as the *CBF/DREB *regulon had been scrutinized in *Arabidopsis*.

**Figure 8:**
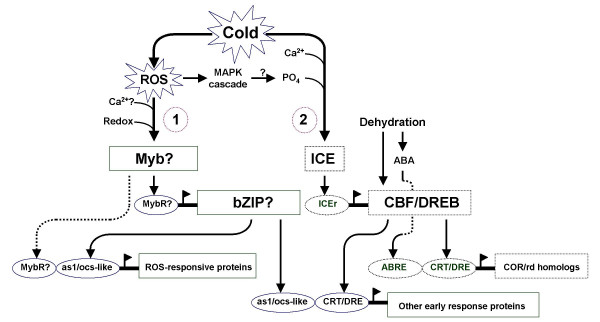
**Hypothetical model of the ROS-mediated gene regulon involved in early responses to chilling in rice and its possible relationship with the *CBF/DREB *and ABA-mediated regulons**. The proposed hierarchical organization of the as1/ocs regulatory cluster is shown in pathway-1. An unknown Myb is expressed constitutively and perhaps activated by change in redox state that is mediated by chilling stress-induced elevation of intracellular ROS. The ROS-activated Myb regulates the transcription of a low temperature-inducible bZIP-type transcription factor that functions as transcriptional activator of as1/ocs-like element-containing genes. A possible alternative or complementary route for regulating the as1/ocs-like element-containing genes is via a constitutively expressed Myb protein (dotted arrow). Pathway-2 shows a general overview of the *CBF/DREB *and ABA-mediated regulons involved in low temperature and dehydration stress response in plants [1, 2, 3, 4]. This hypothetical model proposes that some members of the putative *ROS-bZIP – as1/ocs *regulatory module might also be regulated by *CBF/DREB*.

We assembled a hypothetical model of the ROS-mediated regulon and we propose a hypothesis on its potential relationship with other major regulon(s) involved in low temperature response of the chilling-tolerant japonica rice. Our working hypothesis described in this model is that gene expression during the initial 24 hours at 10°C can be defined (at least partially) in terms of a regulatory cluster (transcription factor-target gene module) that appears to be independent of the *CBF/DREB *and ABA-mediated regulons (Figure 8). The downstream components of this regulatory module (pathway-1 in Figure 8) contain either the prototype as1/ocs element, which is a known target of TGA-type bZIP transcription factors, or the related W-box that is known to bind to TGA-type bZIP and WRKY transcription factors or both. These elements are known to be activated in response to various conditions that trigger the production of excess ROS (particularly H_2_O_2_) including biotic, abiotic and xenobiotic stresses. Genes that contain these promoter elements have also been shown to be induced by exogenously supplied H_2_O_2 _and other chemical signals such as salicylic acid and auxin [31,38,39,50-52].

We also confirmed that a representative subset of '*early response*' genes containing as1/ocs-like promoter elements were responsive to exogenous H_2_O_2_. Based on the percentage of occurrence and enrichment level of the as1/ocs-like elements, we predict that about 80% of the '*early response*' genes that we identified in the semi-global microarray survey are probably responsive to exogenous H_2_O_2 _hence potential members of this regulatory cluster. The specific ferritin gene (Os11g01530) that was profiled in this study was an interesting example of a potential target of regulation by H_2_O_2_. We have shown that this ferritin gene not only contains as1/ocs-like promoter elements but it was also induced by exogenous H_2_O_2_. Ferritin is also directly related by function to H_2_O_2 _metabolism by virtue of its role in the regulation of the Fenton reaction [46]. In addition to ferritin, many of the genes that we identified in this group have also been shown to be responsive to exogenous H_2_O_2 _in *Arabidopsis *[30]. The downstream target cluster of the proposed ROS-mediated regulon also includes a number of transcription factors such as *OsMyb4 *and an EREBP1-type protein, suggesting the possible occurrence of other secondary regulatory clusters under this regulon. This layered organization appears to be consistent with the fact that the ROS-mediated regulon is an '*early response*' mechanism that may be functioning as an initial trigger to a more complex transcriptional network involved in cellular defenses against the physiological injuries caused by chilling.

We hypothesized that a bZIP-type protein is the most likely component of the ROS-mediated pathway upstream to the as1/ocs-like element-containing genes based on the known fact that this family of elements is involved in the recruitment of TGA-type bZIP factors in the promoters of target genes [40]. Based on the composition of the '*early response*' regulatory cluster that we have assembled, this likely regulator is a novel Class-I bZIP protein (*ROS-bZIP1*) that is distinct from the family of known stress-related bZIP transcription factors (Class A). This class of bZIP proteins is quite diverse in function that includes those that are involved in the regulation of vascular development. Recently, some of the members of this class have also been shown to be involved in the development of symptoms to the rice tungro disease [15,53,54]. The significance of this classification with respect to our proposed role of *ROS-bZIP1 *is unclear at this point but can be explained with better understanding or confirmation of the function of *ROS-bZIP1 *during chilling stress.

We have a good rationale to hypothesize that *ROS-bZIP1 *is a likely regulator of the as1/ocs-like element-containing genes based on several peculiar features. First, the timing of expression of *ROS-bZIP1 *is consistent with being an upstream regulator of a cluster of co-regulated genes. For instance, among the '*early response*' transcription factors identified in this study, only *ROS-bZIP1 *showed an induction peak within the initial 2 hours and it was also induced by exogenous H_2_O_2_. The other transcription factors were either induced by chilling at a much later period (same time period as the as1/ocs-like element-containing genes) or not induced by exogenous H_2_O_2 _or both. Based on the differential expression analysis of the chilling-tolerant and intolerant rice cultivars, the timing of expression of the as1/ocs element-containing genes appeared to be a consequence of the early induction of *ROS-bZIP1 *(i.e., *ROS-bZIP1 *precedes the as1/ocs-like element-containing genes). Additionally, we also revisited the original EST libraries used in the assembly of the microarray and we confirmed that *ROS-bZIP1 *and many of the as1/ocs element-containing genes (ferritin, HMG protein, and putative ATP sulfurylase) were co-represented in the same cold stress subtracted EST library but not in the drought stress EST library, consistent with their parallel expression in the microarray. This information provided additional circumstantial evidence in support of our hypothesis that *ROS-bZIP1 *and the as1/ocs element-containing genes are most likely related in a regulatory context. Induction of *ROS-bZIP1 *by exogenous H_2_O_2 _indicates that this gene is a direct target of an ROS-activated upstream component. The second rationale is that *ROS-bZIP1 *is differentially regulated between tolerant and intolerant rice cultivars and its early and robust expression appeared to be positively correlated with chilling tolerance. It appears that timely expression of *ROS-bZIP1 *is one of the initial triggers leading to the activation of the early transcriptome that plays critical roles in the initial physiological adjustments and configures subsequent defenses.

We also acknowledge the possibility that co-expression of *ROS-bZIP1 *and as1/ocs-like element-containing genes could be a mere coincidence, and that they are in fact not directly related in the context of a regulator and downstream target module. This is a possibility that we cannot ignore completely because of the fact that the microarray that we used in the survey consisted of only about 15% of the total genes encoded by the rice genome. There is a possibility that the regulator of the as1/ocs-like element-containing genes was not represented in the miroarray. Our on-going studies that include the analysis of the chilling stress transcriptome using a 45,000-oligonucleotide microarray representing the entire set of rice genes and *in vitro *binding of *ROS-bZIP1 *to the as1/ocs-like sequence will address this issue.

*ROS-bZIP1 *(or any of its homologs in other plants) has not yet been characterized in terms of its potential role in chilling tolerance. With the presumed large size of the putative *ROS-bZIP *– as1/ocs regulatory module, functional analysis of *ROS-bZIP1 *offers an opportunity to reveal another important switch of cold stress gene expression that has not been previously examined in detail in *Arabidopsis*. A cautionary note is that at this point the predicted regulatory relationship between *ROS-bZIP1 *and the as1/ocs element-containing genes awaits further confirmation by promoter deletion-reporter analysis, and direct protein-DNA binding assays such as gel-shift, yeast one-hybrid or chromatin immunoprecipitation. Nevertheless, the regulatory module that we inferred through an integrative genomics approach provides a strong justification for future use of these candidate genes as prototype model for further dissection of the early response ROS-mediated gene regulon by genome-wide transcriptome analysis of transcription factor-overexpressing or knock-out lines.

We found that the upstream regions (-1,000 bp) of the Group-I '*early response*' genes were enriched with potential Myb-target (MybR) motifs. *ROS-bZIP1 *in particular contains three CAAC-type and three other types of MybR motifs, suggesting a possibility that the next component upstream to this gene in the ROS-mediated pathway is probably a Myb-type transcription factor. Constitutively expressed transcription factors could be direct targets of ROS [55]. An H_2_O_2_-sensitive Myb protein is the most likely direct link of the putative *ROS-bZIP – as1/ocs *regulatory module to the chilling induced-H_2_O_2 _signal possibly by activating such protein. Evidence showed that constitutively expressed Myb proteins are activated by redox state-mediated conformational changes in their DNA-binding domains [56]. This could be one possible way by which H_2_O_2 _activates a putative upstream Myb protein that regulates the expression of *ROS-bZIP1*. Alternatively, an inactive Myb protein may be activated through phosphorylation by an upstream kinase that is sensitive to H_2_O_2_. Changes in intracellular Ca^2+ ^concentration may be involved in this activation [57]. Either way, a bZIP-type transcription factor appears to be the most upstream component that is regulated at the transcriptional level by an ROS-activated Myb protein (pathway-1 in Figure 8).

Another interesting feature of the as1/ocs-like element-containing genes is the significant occurrence of MybR motifs (different types combined) in their promoters. For instance, all five genes studied in detail by qPCR including those that were confirmed to be induced by exogenous H_2_O_2 _(*OsMyb4*, EREBP1-type transcription factor, HMG protein, ferritin, and putative ATP sulfurylase) contain different types of MybR motifs. However, unlike the as1/ocs and W-box elements, which are very closely related (nearly identical in sequence), there was no obvious consensus MybR motif found in the promoters of this group of genes. Nevertheless, it is likely that the as1/ocs-like element-containing genes might also be regulated through a secondary route, perhaps involving a possible MybR element and a constitutively expressed but ROS-activated Myb similar to the proposed regulation of *ROS-bZIP1*. In this case, the chilling- and H_2_O_2_-induced expression of this group of genes may be the result of the interaction between as1/ocs-like and certain MybR elements. The slight variation in expression profiles among the Group-II genes examined may be attributable to the potential interaction between as1/ocs-like and certain MybR elements. Alternatively, since all the as1/ocs-like element-containing genes examined by qPCR had significant basal expression level (constitutive level) but highly induced by chilling, the occurrence of certain MybR motifs may be related to a possible modulator function or potential determinants of the basal expression of this specific group of genes.

A number of genes that belong to the proposed *ROS-bZIP – as1/ocs *regulatory module also contain CRT/DRE-like motifs in their promoters. An example of this is ferritin (Os11g01530), which has been reported as a putative *CBF/DREB *target [24]. Previously, we also reported a sequential activation of *CBF/DREB *homolog and CRT/DRE-containing gene (*OsLti6a*) in CT6748-8-CA-17 during the initial 24 hours at 10°C [23]. Both ferritin and *OsLti6a *had genotype-specific expression profiles (CT6748-8-CA-17 vs. INAP12) that were very similar to the patterns of the representative Group-II genes. Therefore, ferritin and *OsLti6a *represent a group of genes that are potentially regulated by both an ROS-responsive bZIP and *CBF/DREB *transcription factors (pathway-2 in Figure 8). This result suggests a potential link between the ROS-mediated and *CBF/DREB *regulons through common downstream target genes. Promoter motif distribution data also showed that less than 40% of the '*early response*' genes identified in this study contain both as1/ocs element and CRT/DRE in their promoters (at least within the -500 region), suggesting that the overlap between these two regulons is probably not that extensive. Comparative microarray analysis of transgenic lines overexpressing *ROS-bZIP1 *and *CBF/DREB *is currently underway to address this hypothesis.

ABA signaling has known linkages with ROS-mediated signaling [41,49]. However, based on our current results it does not appear that the putative *ROS-bZIP – as1/ocs *regulatory module is directly linked to ABA-mediated pathway at least by virtue of the lack of detectable ABA-mediated induction of representative as1/ocs-like element-containing genes. Alternatively, it is possible that the ROS-mediated regulon is upstream to ABA in the signaling pathway. More detailed analysis of the effect of exogenous ABA on a larger set of confirmed H_2_O_2_-responsive genes is required to validate these hypotheses. Genome-wide microarray survey is also currently underway to address this hypothesis.

## Conclusion

Rice is sensitive to even mild cold stress (chilling) particularly at the early stages of seedling establishment. However, japonica cultivars are generally more tolerant than most indica cultivars. Studies aiming to dissect the regulatory networks that define the mechanism by which the responses of a chilling-sensitive plant like rice might differ from the responses of chilling-insensitive plants are just in the early stages. Based on the analysis of a subset of candidate genes, we hypothesized that the early response transcriptome has an important role in the short-term defenses, which are crucial to the survival of a chilling-sensitive plant like rice under above-freezing temperature conditions.

In this study, we have established the rationale that ROS is involved in the early branches of the chilling stress response transcriptional network of rice. We have identified the components of a putative ROS-mediated regulatory module by an integrative analysis of genomics data. We propose that ROS regulates an important component of early response transcriptome by mediating the rapid induction of a group of as1/ocs-like element-containing genes, hence ROS-dependent regulon. We also hypothesized that this regulon is probably independent of the *CBF/DREB *or ABA-mediated regulons in rice. The putative *ROS-bZIP – as1/ocs *regulatory module is currently being validated by biochemical and genetic tests. Given the rapidly growing functional genomics resources for rice, our next step is also to validate the putative regulatory module described in this report with the use of mutants and overexpression lines. Physiological experiments are currently underway to compare the intracellular ROS levels between japonica (chilling tolerant) and indica (chilling intolerant) rice seedlings during the initial 24 hours of chilling.

## Methods

### Plant materials, growth conditions, and stress treatments

Two rice cultivars that exhibit contrasting sensitivity to chilling (CT6748-8-CA-17, tolerant; INIAP12, intolerant) were used in this study [23]. Mature, non-dormant seeds were sterilized in 30% ethanol, rinsed in sterile water and allowed to germinate in 1.5% agar or moist filter paper. Seedlings were allowed to grow to S_3 _stage for 8 to 10 days at 29°C (± 1°C). For the cold stress treatment, S_3_-stage seedlings on agar plates were placed in a growth chamber (Percival Model E-30BHO, Percival Scientifc, Perry, IA) maintained at a constant temperature of 10°C (± 1°C), continuous light (73 μmol sec^-1^m^-2^) and 50–60% RH for the 24 hour duration of the experiment. Control seedlings were grown under the same condition but at 29°C (± 1°C).

ABA and H_2_O_2 _treatments were performed under the same temperature and light conditions as the control experiment. ABA treatment was performed by placing S_3_-stage seedlings on 4 layers of filter paper soaked in 100 μM ABA. H_2_O_2 _treatment was performed by placing S_3_-stage seedlings on four layers of filter paper soaked in 1 mM H_2_O_2 _for 2 hours and then transferring them back to layers of water soaked filter paper for another 10 hours (pulse-treatment). In both ABA and H_2_O_2 _experiments, control seedlings were grown on four layers of water-soaked filter paper. Total RNA was isolated from frozen shoot tissues with the TRIzol reagent (Invitrogen, Grand Island, NY) following the manufacturer's protocol.

### Microarray analysis

A cDNA microarray was assembled by the Michigan State University Genomics Technology Support Facility (East Lansing, MI). This microarray contains 5,855 features derived from a unigene set of a subtracted EST library of cold stressed rice at S_3 _seedling stage and a normalized EST library of drought stressed rice plants at booting stage [29]. Individual microarray features were spotted in duplicate, hence each slide consists of 11,710 spots (technical replicate) representing 5,855 unique features. The target RNA samples for microarray hybridization were from seedlings (S_3 _stage) of the chilling tolerant cultivar CT6748-8-CA-17 after 0.5, 2, 6, 12 and 24 hours at 10°C. Separate control RNA from seedlings maintained at 29°C was used for each stress time point to cancel out potential developmental effects between control and chilling stressed seedlings.

Two independent biological replicates were performed for each pair of control and chilling stress treatments. Equal amounts of total RNA (20 μg) from chilling stressed and control seedlings were reverse transcribed and labeled with Cy3 and Cy5 dyes, respectively, using the CyScribe post-cDNA synthesis labeling kit (Amersham-GE Healthcare, Piscataway, NJ) according to the manufacturer's protocol. Microarray slides were hybridized for 16 hr in humidified chambers maintained at 42°C. Following hybridization, the microarray slides were subjected to three-step stringency washes (2X SSC + 1% sarcosyl, 2X SSC, 0.2X SSC). Gene expression data was acquired by measuring the fluorescence intensity of each spot at 532 nm (Cy3) and 635 nm (Cy5) with the GenePix Pro 4200 scanner (Axon Instruments, Union City, CA). The signal intensity of each spot was expressed as medians of pixels after background subtraction, i.e., signal minus background (F_635 _or F_532_) Median – B_635 _or B_532_). Expression values were normalized so that mean of the ratio of medians of all of the features is equal to 1. This normalization method is based on the premise that most genes on the array are not differentially expressed, and therefore the arithmetic mean of the ratios from every feature on a given array should be equal to 1. Microarray data can be accessed at the Gene Expression Omnibus (Acc. GSE7071).

Microarray data was analyzed using the Acuity Bioinformatics Suite (Axon Instruments, Union City, CA). The average signal intensity values of the duplicate spots were averaged between biological replicates and values were expressed as a base_2 _logarithm of the ratio of medians, i.e., log_2 _(F_532_median-B_532_)/(F_635_median-B_635_). Log ratios were transformed to fold-change values in subsequent analyses. Significant change in gene expression was defined as fold-increase in chilling stress relative to control at or above a cut-off level of 1.8-fold.

### *Ab initio *analysis of promoters

ESTs for all upregulated microarray features were anchored to the map-based sequence of japonica rice using the TIGR rice genome browser. The designated genomic ORF for a feature was identified based on the closest match in blastN alignment (lowest e-value; in most cases this is equal to or very close to 0), hence, features were subsequently designated by the locus number of the corresponding genomic ORF. Based on the most current genome annotation, the transcription start site (TSS), initiation codon and predicted TATA box of a given locus were determined. The upstream region of a genomic locus from the start codon was extracted from the genome sequence and used for subsequent analysis. Our analysis of the sequences of few CT6748-8-CA-17 genomic clones of Group-II genes showed identical sequences with the Nipponbare reference.

Significantly overrepresented promoter motifs were identified using an *ab initio *method implemented in the Dragon Motif Builder program with EM1 and EM2 option [33]. With the EM1 option the system attempts to extend or shrink motifs to find the best motif length for a family, while in the EM2 option all possible motif lengths within certain range are examined and the best collections of motifs are selected. Up to 30 motif families were detected in each run using motif length in the range of 5–10 nucleotides. The threshold values used for detection were set at 0.85, 0.875, 0.90 and 0.95 from the range of (0, 1). Since the identification of motif families is heuristic and influenced by the overall DNA sequence composition, the variations in the length of the searched regions may influence the type of motifs identified. As we had no prior information where the motifs of interest could be located, we experimented with different lengths of promoters covering (-1000, -1), (-1000, +350), (-500, -1), (-500, +350) and (-500, +200) relative to the TSS. Details of motif families identified are given in the Table 2.

To enhance the detection of stress-related motif families, promoter sequences of genes not associated with cold stress were selected at random and used as background or negative control dataset. The biological significance of the identified motifs was inferred by their presence in databases such as PLACE [34], TRANSFAC [35], and AGRIS [36]. Enrichment levels of the candidate motifs were based on the e-value and percentage of occurrence relative to the background sequence. The homogeneity of the motifs in each individual group (total information content) is related to the average binding energy for the collection of sites [58]. The motifs predicted using this scheme were also compared with the results of similar analysis using the MEME/MAST System for motif discovery [37].

### RNA gel-blot analysis

Equal amounts (20 μg) of total RNA samples from control and ABA-treated seedlings were fractionated by electrophoresis in formaldehyde gels (Ambion, Austin, TX). The RNA was blotted onto Hybond N^+ ^nylon membrane (Amersham-GE Healthcare, Piscataway, NJ) using standard procedures. EST probes were random-prime labeled with α^32^P-dCTP using the RediPrime kit (Amersham-GE Healthcare, Piscataway, NJ). RNA gel blot was hybridized with ^32^P-labeled probes (Amersham-GE Healthcare, Piscataway, NJ) by standard procedures.

### Real-time PCR (qPCR) analysis

Total RNA (1 μg) was reverse transcribed using oligo-dT and random primer cocktail and iScript cDNA Synthesis kit (Bio-Rad Laboratories, Hercules, CA) following the manufacturer's instructions. Gene-specific primers for the control (actin, Os10g36650) and experimental genes were designed based on the sequences of corresponding genomic ORF (Table 3). The actin gene was chosen as the reference based on its constitutive expression in the microarray and RNA gel blot data. The actin-specific primers were designed to span an exon interrupted by 100-bp intron. Thus, amplified actin fragments from cDNA and genomic DNA templates were 120 bp and 220 bp, respectively. This method allowed effective use of the control actin gene to assess background amplification from genomic DNA contamination.

**Table 3:** Gene-specific primers used for quantitative RT-PCR analysis. Primers were designed based on EST and corresponding genomic locus.

**Primer name**	**Sequence (5' to 3')**	**Locus No.**
CAT1-F	CCGTATGGAACAACAACAACTC	Os03g03910
CAT1-R	GATACGCTCCCTGTCGAAGT	
bZIP-F	AGCTAAGAGATGCCCTGAATGAAG	Os08g43090
bZIP-R	TCTGCGATGACTGTTGTTGCTGTAT	
bHLH-F	CGGGATCGAGCAGGCGGTCAT	Os01g70310
bHLH-R	GCAGGAGCACGGTCTTAATTTCTTCAGG	
Myb-F	CCAAGGAGGAGGAGGACACCATC	Os04g43680
Myb-R	GCATCGAGGCGCTTCTTGAGG	
HMG-F	GGAAGGCCGGCAAGGACCCCAACA	Os06g51220
HMG-R	TTCCACCTATCACCGGCTGCTTTTCCTACA	
ATPSul-F	GCCCAGCTGCGTGAAGAGTTTG	Os03g53230
ATPSul-R	AAGAAGGCGTTTGCGTGTATCAGTCA	
Ferri-F	CCCCCAGGCCAAGGACCAGT	Os11g01530
Ferri-R	ACGATCAAAGTAGGCGAAAAGGGAGTG	
DI19-F	GGGTGCCCATTTCAGAGTTCA	Os01g73960
DI19-R	GCAGATAAATTGGGAGAGCAGTGG	
ATPSyn-F	AGCTCGTGATGAAGTCCATCGTGC	Os12g07140
ATPSyn-R	CGTGCTGATGATGACTGCGATGAT	
AUX-F	CAAGAACACGATGGCAACCAACCA	Os01g13030
AUX-R	TTACCGGTGCTGAAGCCAATGAAC	
Pfkin-F	CCAAGCCGGGCAGCCTCTCGTT	Os01g47550
Pfkin-R	CGCGCCGCCACCGTCTCC	
Ripe-F	CTGTGTTTCGCCTTCGTCTACCATCT	Os04g30490
Ripe-R	TCTGCGGGCAAGGACGAACTG	
AnTr-F	TCTCTGAACCCGGATGATATGGACTGC	Os03g05390
AnTr-R	CGATGCCCGGAGCGACTTGAT	
ACT2-F	CATGAAGATCAAGGTTGTCGCTCC	Os10g36650
ACT2-R	CCAGATTCTTCATACTCAGCCCTTG	
EREBP1-F	CTGCCAAAAGAAAGAGAAAGAACCAAT	Os02g54160
EREBP1-R	TGCCAAGCCAGACACGAACAC	

Quantitative RT-PCR (qPCR) analysis of the control (actin) and experimental genes were performed in at least three independent replicates using a single color real-time PCR system with SYBR Green Supermix (Bio-Rad Laboratories, Hercules, CA). Each qPCR cocktail consisted of the cDNA template (2 μl) and gene-specific oligonucleotide primers (4 ng/μl) in a total reaction volume of 20 μl. The cycling parameters were as follows: cDNA template denaturation at 94°C (20 sec), annealing at primer-specific temperature (15 sec), extension at 72°C (20 sec) for a total of cycles, followed by a final extension step at 72°C (5 min). The qPCR profiles of experimental genes were normalized relative to the control actin gene expression. The relative expression of each experimental gene was based on ΔC_T _normalized against the reference actin gene (Bio-Rad PCR Manual, Bio-Rad Laboratories, Hercules, CA). The relative expression values of the control (time point 0 hour) was set to zero by subtracting the values from the treatment values at all time points.

## Authors' contributions

CC performed the entire microarray experiments including analysis, RNA gel blot analyses, anchoring of ESTs to the rice genome sequence and manual analysis of promoter sequences. KYY designed and performed all qPCR analyses, manual promoter analysis, anchoring of ESTs to the genome sequence, submitted the microarray data to GEO and contributed in the assembly of the hypothetical model of ROS-mediated regulon. HR contributed to the analysis of microarray data. YJ and SJY helped in the assembly of unigenes for cDNA microarray. BM and VBB designed and performed the *ab initio *promoter analysis by the Dragon Motif Builder. BGDR conceptualized, designed and coordinated the whole study, constructed and analyzed the subtracted EST library and assembled the unigene set for the cDNA microarray. He also performed the integrated analysis of the transcriptome and promoter data, assembled the hypothetical model for the ROS-mediated regulon and wrote the entire manuscript. All authors read and approved the final manuscript.

## Supplementary Material

Additional file 1 Genes with ≥ 1.8-fold induction during the initial 24 hr of exposure at 10°C. This table provides a list of all the genes identified from the microarray survey that exhibited at least 1.8-fold induction in at least one time point during the initial 24 hr of chilling stress. The corresponding genomic locus number (TIGR rice genome annotation) and temporal grouping (Group 1 or Group II) are also indicated. Click here for file

Additional file 2 Time-course profiles of 121 '*early response*' genes. The temporal expression patterns of all identified upregulated genes are summarized in this table with fold-change values. Click here for file

## References

[B1] Benedict C, Geisler M, Trygg J, Huner N, Hurry V (2006). Consensus by democracy. Using meta-analyses of microarray and genomic data to model the cold acclimation signaling pathway in *Arabidopsis *. Plant Physiol.

[B2] Chinnusamy V, Zhu J, Zhu JK (2006). Gene regulation during cold acclimation in plants. Physiol Plant.

[B3] Nakashima K, Yamaguchi-Shinozaki K (2006). Regulons involved in osmotic stress-responsive and cold stress-responsive gene expression in plants. Physiol Plant.

[B4] Van Buskirk H, Thomashow MF (2006). *Arabidopsis *transcription factors regulating cold acclimation. Physiol Plant.

[B5] Nordin K, Vahala T, Palva ET (1993). Differential expression of two related low temperature induced genes of *Arabidopsis thaliana *. Plant Mol Biol.

[B6] Shinozaki K, Yamaguchi-Shinozaki K (2000). Differences and cross-talk between two stress signaling pathways. Curr Opin Plant Biol.

[B7] Thomashow MF (1999). Freezing tolerance genes and regulatory mechanisms. Ann Rev Plant Physiol Plant Molec Biol.

[B8] Yamaguchi-Shinozaki K, Shinozaki K (1993). Characterization of the expression of a desiccation-responsive *rd29 *gene of *Arabidopsis thaliana *and analysis of its promoter in transgenic plants. Mol Gen Genet.

[B9] Artus NN, Uemura M, Steponkus PL, Gilmour SJ, Lin CT, Thomashow MF (1996). Constitutive expression of the cold-regulated *Arabidopsis thaliana COR15a *gene affects both chloroplast and protoplast freezing tolerance. Proc Natl Acad Sci USA.

[B10] Cook D, Fowler S, Fiehn O, Thomashow MF (2004). A prominent role for the CBF cold response pathway in configuring the low temperature metabolome of *Arabidopsis *. Proc Natl Acad Sci USA.

[B11] Gilmour SJ, Sebolt AM, Salazar MP, Everard JD, Thomashow MF (2000). Overexpression of the *Arabidopsis CBF3 *transcriptional activator mimics multiple biochemical changes associated with cold acclimation. Plant Physiol.

[B12] Liu Q, Kasuga M, Sakuma Y, Abe H, Miura S, Yamaguchi-Shinozaki K, Shinozaki K (1998). Two transcription factors, *DREB1 *and *DREB2*, with an EREBP/AP2 DNA binding domain separate two cellular signal transduction pathways in drought- and low temperature-responsive gene expression, respectively in *Arabidopsis*. Plant Cell.

[B13] Fowler S, Thomashow MF (2002). *Arabidopsis *transcriptome profiling indicates that multiple regulatory pathways are activated during cold acclimation in addition to the *CBF *cold response pathway. Plant Cell.

[B14] Giraudat J, Parcy F, Bertandre N, Gosti F, Leung J, Morris PC, Bouvier-Durand M, Vartanian N (1994). Current advances in abscisic acid action and signaling. Plant Mol Biol.

[B15] Jakoby M, Weisshaar B, Droge-Laser W, Vicente-Carbajosa J, Tiedemann J, Kroj T, Parcy F (2002). bZIP transcription factors in *Arabidopsis *. Trends Plant Sci.

[B16] Reichmann JL, Meyerowitz EM (1998). The AP2/EREBP family of plant transcription factors. Biol Chem.

[B17] Stockinger EJ, Gilmour SJ, Thomashow MF (1997). *Arabidopsis thaliana CBF1 *encodes an AP2 domain-containing transcription activator element that binds to the C-repeat/DRE, a cis-acting regulatory element that stimulates transcription in response to low temperature and water deficit. Proc Natl Acad Sci USA.

[B18] Jaglo-Ottosen KR, Gilmour SJ, Zarka DG, Schabenberger O, Thomashow MF (1998). *Arabidopsis CBF1 *overexpression induces *COR *genes and enhances freezing tolerance. Science.

[B19] Kasuga M, Kiu Q, Miura S, Yamaguchi-Shinozaki K, Shinozaki K (1999). Improving plant drought, salt and freezing tolerance by gene transfer of a single stress-inducible transcription factor. Nature Biotech.

[B20] Vogel JT, Zarka DG, Van Buskirk H, Fowler SG, Thomashow MF (2005). Roles of the *CBF2 *and *ZAT12 *transcription factors in configuring the low temperature transcriptome of *Arabidopsis*. Plant J.

[B21] Lee B, Henderson DA, Zhu JK (2005). The *Arabidopsis *cold-responsive transcriptome and its regulation by *ICE1*. Plant Cell.

[B22] Lee H, Guo Y, Ohta M, Xiong L, Stevenson B, Zhu JK (2002). *LOS2*, a genetic locus required for cold-responsive gene transcription encodes a bi-functional enolase. EMBO J.

[B23] Morsy MR, Almutairi AM, Gibbons J, Yun SJ, De los Reyes BG (2005). The *OsLti6 *genes encoding low-molecular-weight membrane proteins are differentially expressed in rice cultivars with contrasting sensitivity to low temperature. Gene.

[B24] Rabbani MA, Maruyama K, Abe H, Khan MA, Katsura K, Ito Y, Yoshiwara K, Seki M, Shinozaki K, Yamaguchi-Shinozaki K (2003). Monitoring expression profiles of rice genes under cold, drought, and high salinity stresses and abscisic acid application using cDNA microarray and RNA gel-blot analyses. Plant Physiol.

[B25] Yazaki J, Shimatani Z, Hashimoto A, Nagata Y, Fujii F, Kojima K, Suzuki K, Taya T, Tonouchi M, Nelson C, Nakagawa A, Otomo Y, Murakami K, Matsubara K, Kawai J, Carninci P, Hayashizaki Y, Kikuchi S (2004). Transcriptional profiling of genes responsive to abscisic acid and gibberellin in rice: phenotyping and comparative analysis between rice and *Arabidopsis *. Physiol Genomics.

[B26] Zhang X, Fowler SG, Cheng H, Lou Y, Rhee SY, Stockinger EJ, Thomashow MF (2004). Freezing-sensitive tomato has a functional CBF cold response pathway, but a *CBF *regulon that differs from that of freezing-tolerant *Arabidopsis*. Plant J.

[B27] Dubouzet JG, Sakuma Y, Ito Y, Kasuga M, Dubouzet EG, Miura S, Seki M, Shinozaki K, Yamaguchi-Shinozaki Y (2003). *OsDREB *genes in rice, *Oryza sativa *L., encode transcription activators that function in drought-, high salt-, and cold-responsive gene expression. Plant J.

[B28] Provart NJ, Gil P, Chen W, Han B, Chang HS, Wang X, Zhu T (2003). Gene expression phenotypes of *Arabidopsis *associated with sensitivity to low temperatures. Plant Physiol.

[B29] De los Reyes BG, Morsy M, Gibbons J, Varma TSN, Antoine W, McGrath JM, Halgren R, Redus M (2003). A snapshot of the cold stress transcriptome of developing rice seedlings (*Oryza sativa *L.) via ESTs from subtracted cDNA library. Theor Appl Genet.

[B30] Desikan R, Mackerness SAH, Hancock JT, Neill SJ (2001). Regulation of the Arabidopsis transcriptome by oxidative stress. Plant Physiol.

[B31] Ulker B, Somssich IE (2004). WRKY transcription factors: from DNA binding towards biological function. Cur Op Plant Biol.

[B32] Vaninni C, Locatelli F, Bracale M, Magnani E, Marsoni M, Osnato M, Mattana M, Baldoni E, Coraggio I (2004). Overexpression of the rice *Osmyb4 *gene increases chilling and freezing tolerance of *Arabidopsis *thaliana plants. Plant J.

[B33] Huang L, Yang R, Chowdhary A, Kassim V, Bajic VB (2005). An algorithm for *ab initio *DNA motif detection. Information Processing and Living Systems, World Scientific.

[B34] Higo K, Ugawa Y, Iwamoto M, Korenaga T (1999). Plant cis-acting regulatory DNA elements (PLACE) database. Nucl Acids Res.

[B35] Matys V, Fricke E, Geffers R, GoBling E, Haubrock M, Hehl R, Hornischer K, Karas D, Kel AE, Kel-Margoulis OV, Kloos DU, Land S, Lewicki-Potapov B, Michael H, Munch R, Reuter I, Rotert S, Saxel H, Scheer M, Thiele S, Wingender E (2003). TRANSFAC: transcriptional regulation, from patterns to profiles. Nucl Acids Res.

[B36] Davuluri RV, Sun H, Palaniswamy SK, Matthews N, Molina C, Kurtz M, Grotewold E (2003). *Arabidopsis *AGRIS: Gene Regulatory Information Server, an information resource of Arabidopsis cis-regulatory elements and transcription factors. BMC Bioinformatics.

[B37] Bailey TL, Williams N, Misleh C, Li WW (2006). Discovering and analyzing DNA and protein sequence motifs. Nucl Acids Res.

[B38] Chen W, Singh KB (1999). The auxin, hydrogen peroxide and salicylic acid induced expression of the *Arabidopsis GST6 *promoter is mediated in part by an ocs element. Plant J.

[B39] Garreton V, Capinelli J, Jordana X, Holuigue L (2002). The as-1 promoter element is an oxidative stress-responsive element and salicylic acid activates it via oxidative species. Plant Physiol.

[B40] Johnson C, Broden E, Arias J (2003). Salicylic acid and *NPR1 *induce the recruitment of trans-activating TGA factors to a defense gene promoter in *Arabidopsis*. Plant Cell.

[B41] Orozco-Cardenas ML, Narvaez-Vasquez J, Ryan CA (2001). Hydrogen peroxide acts as a secondary messenger for the induction of defense genes in tomato plants in response to wounding, systemin, and methyl jasmonate. Plant Cell.

[B42] Bolwell GP, Bindschendler LV, Blee KA, Butt VS, Davies DR, Gardner SL, Gerrish C, Minibayeva F (2002). The apoplastic oxidative burst in response to biotic stress in plants: a three-component system. J Expt Bot.

[B43] Corpas FJ, Barroso JB, del Rio LA (2001). Peroxisomes as a source of reactive oxygen species and nitric oxide signal molecules in plant cells. Trends Plant Sci.

[B44] Neill S, Desikan R, Hancock J (2002). Hydrogen peroxide signaling. Cur Op Plant Biol.

[B45] Sagi M, Fluhr R (2006). Production of reactive oxygen species by plant NADPH oxidases. Plant Physiol.

[B46] Hegedus A, Erdei S, Janda T, Szalai J, Dudits D, Horvath G (2002). Effects of low temperature stress on ferritin or aldose reducatse overexpressing transgenic tobacco plants. Acta Biol Sze.

[B47] Finkel T (2000). Redox-dependent signal transduction. FEBS Lett.

[B48] Kovtun Y, Chiu WL, Tena G, Sheen J (2000). Functional analysis of oxidative stress-activated mitogen-activated protein kinse cascade in plants. Proc Natl Acad Sci USA.

[B49] Zhang X, Zhang L, Dong F, Gao J, Galbraith DW, Song CP (2001). Hydrogen peroxide is involved in abscisic acid-induced stomatal closure in *Vicia faba *. Plant Physiol.

[B50] Lebel E, Heifetz P, Thorne L, Uknes S, Ryals J, Ward E (1998). Functional analysis of regulatory sequences controlling *PR-1 *gene expression in *Arabidopsis*. Plant J.

[B51] Mare C, Mazzucotelli E, Crosatti C, Francia E, Stanca AM, Cattivelli L (2004). *Hv-WRKY38 *: a new transcription factor involved in cold- and drought-response in barley. Plant Mol Biol.

[B52] Zhang Y, Tessaro MJ, Lassner M, Li X (2003). Knockout analysis of *Arabidopsis *transcription factors TGA2, TGA5, and TGA6 reveals their redundant and essential roles in systemic acquired resistance. Plant Cell.

[B53] Dai S, Zhang Z, Chen S, Beachy RN (2004). *RF2b*, a rice bZIP transcription activator, interacts with *RF2a *and is involved in symptom development of rice tungro disease. Proc Natl Acad Sci USA.

[B54] Yin Y, Zhu Q, Dai S, Lamb C, Beachy RN (1997). *RF2a*, a bZIP transcriptional activator of the phloem-specific rice tungro bacilliform virus promoter, functions in vascular development. EMBO J.

[B55] Delaunay A, Isnard AD, Toledano MB (2000). H_2_O_2 _sensing through oxidation of the *Yap1 *transcription factor. EMBO J.

[B56] Myrset AH, Bostard A, Jamin N, Lirsac PN, Toma F, Gabrielsen OS (1993). DNA and redox state induced conformational changes in the DNA-binding domain of the Myb oncoprotein. EMBO J.

[B57] Knight H, Trewavas AJ, Knight MR (1996). Cold calcium signaling in *Arabidopsis *involves two cellular pools and a change in calcium signature after acclimation. Plant Cell.

[B58] Stormo GD (2000). DNA binding sites: representation and discovery. Bioinformatics.

