# Supplementary material for: An early response regulatory cluster induced by low temperature and hydrogen peroxide in seedlings of chilling-tolerant japonica rice
Source: BMC Genomics. 2007 Jun 18;8:175. doi: 10.1186/1471-2164-8-175 (PMC1925099; doi:10.1186/1471-2164-8-175)
Supplement: Additional file 1 — Genes with ≥ 1.8-fold induction during the initial 24 hr of exposure at 10°C. This table provides a list of all the genes identified from the microarray survey that exhibited at least 1.8-fold induction in at least one time point during the initial 24 hr of chilling stress. The corresponding genomic locus number (TIGR rice genome annotation) and temporal grouping (Group 1 or Group II) are also indicated. [file 1471-2164-8-175-S1.doc]

Additional file 1: Genes with >1.8-fold induction during the initial 24 hr at 10^o^C.

| **EST annotation^a^** | **EST Accession** | **Locus No.** |
| --- | --- | --- |
| **Group-I** |  |  |
| Similar to Arabidopsis expressed protein | CA763335 | Os12g19350 |
| Putative malate dehydrogenase | CA998008 | Os01g46070 |
| Putative ubiquitin conjugating enzyme | CA998024 | Os10g11260 |
| Hypothetical protein | CA759740 | unknown |
| Channel protein (MIP) similar to NOD26 | CA761959 | Os06g12310^b^ |
| Hypothetical protein | CA765797 | Os02g42540 |
| Mitochondrial processing peptidase α-II chain | CA999664 | Os01g09560 |
| GDP dissociation inhibitor protein**^c^** | CA766013 | Os05g34540 |
| Rice impaired sucrose induction protein | CA998323 | Os06g03850 |
| Unknown rice protein | CA998424 | Os03g27820 |
| α-Amylase isozyme-3E precursor | CA999235 | Os08g36900 |
| **bHLH protein** | **CA998014** | **Os01g70310**^d^ |
| **bZIP transcription factor (*ROS-bZIP1*)** | **CA998073** | **Os08g43090**^bce^ |
| Universal stress protein, expressed | CA997955 | Os05g37970 |
| S-adenosylmethionine synthetase-1 | CA998007 | Os05g04510 |
| Similar to Arabidopsis avrRpt2-related protein | CA999193 | Os02g04800 |
| Harpin-induced gene-1 homolog | CA999922 | Os04g58850 |
| Putative casein kinase-II beta subunit | CB000323 | Os10g41520 |
| Similar to Arabidopsis vacuolar sorting protein | CA998160 | Os11g41130^b^ |
| Proteasome subunit alpha type-2 | CA998038 | Os02g42320 |
| **Group-II** |  |  |
| Putative guanine deaminase | CA759494 | Os07g14150 |
| Putative glutamate dehydrogenase | CA760006 | Os02g43470 |
| **Putative ATP sulfurylase** | **CB000478** | **Os03g53230**^bd^ |
| Elongation factor 1α (EF-1α) | CA997866 | Os03g08050 |
| 26S proteasome regulatory AAA-ATPase subunit-6 | CA998999 | Os02g11050 |
| Fatty acid beta oxidation multifunctional protein | CA998085 | Os02g17390 |
| Auxin-induced protein AUX28 | CA998153 | Os12g40890^c^ |
| Proteasome α-subunit | CA998346 | Os08g43540 |
| Glycine-rich cell wall structural protein | CA998351 | Os03g14650 |
| Ankyrin-like protein^d^ | CA766136 | Os01g61990 |
| Non-specific lipid transfer protein | CB000626 | Os12g02320 |
| pfkb-kinase domain protein | CA998167 | Os01g47550^bd^ |
| **Putative auxin-responsive protein IAA8** | **CB000117** | **Os01g13030**^c^ |
| Unknown expressed protein | CA999155 | Os01g14690 |
| Acetyl-CoA synthetase | EL586673 | unknown |
| Auxin-responsive protein IAA9 | CA999284 | Os05g14180^c^ |
| Pyruvate kinase, cytosolic isozyme | CA999799 | Os04g58110 |
| Phenylalanine ammonia lyase | CA998134 | Os04g43800^c^ |
| Putative HAK2 potassium ion transporter | CA998043 | Os01g70940 |

**^a^** Annotation based on the most significant BlastN hit. **^b^**Microarray data validated by northern blot (data not shown).

**^c^** Putative homologs of Arabidopsis H_2_O_2_-responsive genes [30]. **^d^**Genes analyzed by quantitative PCR.

Additional file 1 cont’d.

| **EST annotation^a^** | **EST Accession** | **Locus No.** |
| --- | --- | --- |
| **Group II** (cont’d) |  |  |
| Oxysterol-binding protein | CA998055 | Os03g16690 |
| Putative plastidic cysteine synthase-1 | CA999320 | Os01g74650 |
| Putative AMP-binding protein | CA998788 | Os03g04000 |
| Similar to two-component response-like protein PRR95 | CA764459 | Os09g36220 |
| 60S Ribosomal protein L10-3 | CB000149 | Os05g07700 |
| Putative anion transporter | CA759231 | Os03g05390^bc^ |
| Phosphoesterase family protein | CA759247 | Os03g61130^b^ |
| 40S ribosomal protein S3A | CA759562 | Os12g21798 |
| NADH-glutamate synthase | CA760007 | Os01g48960 |
| *O. sativa* ORF_OSJNBa0047D12.20 | CA760100 | unknown |
| Putative 30S ribosomal protein L13 | CA763167 | Os05g15370 |
| Drought-induced protein-19 | CA766210 | Os01g73960 |
| Putative ripening regulated protein | CA766427 | Os04g30490 |
| ADP-ribosylation factor | CB096284 | Os01g16030 |
| Putative serine/threonine protein kinase | CB096630 | Os07g48100 |
| **Ethylene response element binding protein (EREBP1)** | **CB096828** | **Os02g54160**^bd^ |
| Similar to a reversibly glycosylated protein | CA997913 | Os04g56520 |
| Sec61p transfer protein | CA997842 | Os09g17830 |
| Malate dehydrogenase, cytoplasmic | CA997843 | Os10g33800 |
| Enolase | CA997931 | Os10g08550^b^ |
| **High mobility group protein (HMG)** | **CA997954** | **Os06g51220**^bd^ |
| **Ferritin** | **CA999789** | **Os11g01530**^bcd^ |
| Glycosyl hydrolase family-1 protein | CA999930 | Os03g49600 |
| Mannose 6-phosphate isomerase | CA999612 | Os11g38810 |
| Putative ethylene forming enzyme | CB000008 | Os03g32470 |
| Putative disulfide isomerase | CB000189 | Os05g06430 |
| Putative serine hydroxymethyltransferase | CA765451 | Os05g35440 |
| Disease resistance protein (sumoylation ligase E3) | CA765471 | Os07g25890 |
| Protein disulfide isomerase | CB000459 | Os11g09280 |
| Glyceraldehyde 3-phosphate dehydrogenase, cytosolic | CA997943 | Os08g03290 |
| *O. sativa* ORF_OSJNBa0047D12.20 | CA760100 | unknown |
| Ligand-gated ion channel | CA760356 | Os06g09120 |
| Putative cullin | CA764973 | Os01g27150 |
| Polyubiquitin | CA998086 | Os06g46770 |
| Rab-GDP dissociation inhibitor protein | CB096560 | Os05g23860^b^ |
| 2-Oxoglutarate/malate translocator protein | CB096933 | Os05g11780 |
| Geranyl diphosphate synthase | CB097201 | Os05g50550 |
| Polyubiquitin | CA767055 | Os05g42424 |
| Putative 60S ribosomal protein L7 | CA997837 | Os08g13690 |
| Glyceraldehyde 3-phosphate dehydrogenase | CA997870 | Os02g38920 |
| Expressed protein ORF_P0501G01.5 | CA997862 | Os01g43370 |
| Phenylalanine ammonia lyase | CA997981 | Os02g41630^c^ |

**^a^** Annotation based on the most significant BlastN hit. **^b^**Microarray data validated by northern blot (data not shown).

**^c^** Putative homologs of Arabidopsis H_2_O_2_-responsive genes [30]. **^d^**Genes analyzed by quantitative PCR.

Additional file 1 cont’d.

| **EST annotation^a^** | **EST Accession** | **Locus No.** |
| --- | --- | --- |
| **Group II** (cont’d) |  |  |
| Putative 60S ribosomal protein | CA999946 | Os08g44450 |
| Phosphoglycerate kinase, cytosolic | CA998447 | Os02g07260 |
| **Myb protein (*OsMyb4*)** | **CB000724** | **Os04g43680**^bcd^ |
| Smr domain-containing protein | CA999596 | Os06g09890^b^ |
| Putative cellulose synthase-8 | CB000645 | Os07g14850 |
| Bisphosphoglycerate-indep. phosphoglycerate mutase | CA999846 | Os01g60190 |
| Probable inorganic disphosphatase | CB000017 | Os02g55890 |
| NB-ARC domain containing protein | CB000495 | Os11g13940 |
| Expressed protein AAP54340.1 | CA764838 | Os10g34370 |
| Potassium (K^+^) transport related protein | EL586674 | unknown |
| 40S Ribosomal protein S9 | CA997973 | Os03g05980 |
| 40S Ribosomal protein S4 | CB000020 | Os02g01560 |
| Similar to an integral membrane protein-like | CB001067 | Os07g26110 |
| Ribosomal protein S19 | CA998493 | Os04g16780 |
| Ribosomal protein S15 | CB000751 | Os07g10720 |
| **Expressed protein (ATP synthase-like)** | **CA997967** | **Os12g07140**^bd^ |
| SF21-like protein | CA760250 | Os01g56590 |
| Citrate synthase | CA998959 | Os02g10070 |
| Ubiquitin/ribosomal protein S27a | CA764642 | Os01g22490 |
| Copper chaperone/heavy metal associated domain | CB000247 | Os02g32814 |
| Similar to Arabidopsis expressed protein | CA763996 | Os02g57750 |
| α-Amylase-catalytic domain containing protein | CA998461 | Os06g26234 |
| Phospholipid transfer protein homolog | CA998474 | Os11g24070 |
| Expressed ZIM motif family protein | CA999670 | Os07g42370^b^ |
| Phosphatidylinositol transfer protein III | CA759466 | Os02g04020 |
| Aldehyde dehydrogenase | CA761977 | Os11g08300 |
| Putative ADP ribosylation factor | CA762909 | Os01g59790 |
| Similar to early nodulin OsENOD93a protein | CA763224 | Os06g04930 |
| Putative leucine-rich repeat (LRR) protein | CA767317 | Os03g32580^b^ |
| 40S ribosomal protein | CA999633 | Os03g10340 |
| Similar to Arabidopsis protein At3g51050 | CA766064 | Os02g01070 |
| DNAJ-like protein | CA999019 | Os02g43930 |
| Putative transaldolase | CA998060 | Os01g70170 |
| C3HC4-type ring finger protein | CA998362 | Os02g36740 |
| Similar to an unknown Arabidopsis protein | CA763291 | Os03g16800 |
| GDP dissociation inhibitor protein | CA766013 | Os05g34540^b^ |
| Putative splicing factor-like protein | CA763100 | Os10g30370 |
| Cyclophilin | CA767313 | Os06g49480 |
| Nucleic acid binding protein with RNA recognition motif | CA762747 | Os02g35950 |
| 26S proteasome non-ATPase subunit-5 | CA759543 | Os03g63430 |

**^a^** Annotation based on the most significant BlastN hit. **^b^**Microarray data validated by northern blot (data not shown).

**^c^** Putative homologs of Arabidopsis H_2_O_2_-responsive genes [30]. **^d^**Genes analyzed by quantitative PCR.
