# Supplementary material for: An early response regulatory cluster induced by low temperature and hydrogen peroxide in seedlings of chilling-tolerant japonica rice
Source: BMC Genomics. 2007 Jun 18;8:175. doi: 10.1186/1471-2164-8-175 (PMC1925099; doi:10.1186/1471-2164-8-175)
Supplement: Additional file 2 — Time-course profiles of 121 'early response' genes. The temporal expression patterns of all identified upregulated genes are summarized in this table with fold-change values. [file 1471-2164-8-175-S2.doc]

Additional file 2: Time-course profiles of 121 ‘*early response*’ genes.

| **EST Acc.** | **Locus No.** | **0.5 hr** | **2 hr** | **6 hr** | **12 hr** | **24 hr** |
| --- | --- | --- | --- | --- | --- | --- |
| CA759231 | LOC_Os03g05390 | -1.247 | -1.378 | 1.223 | 2.157 | 2.329 |
| CA759247 | LOC_Os03g61130 | 1.081 | -1.117 | 1.408 | 2.385 | 2.866 |
| CA759466 | LOC_Os02g04020 | -1.181 | -1.188 | -1.044 | 1.356 | 2.329 |
| CA759494 | LOC_Os07g14150 | -1.057 | 1.046 | 1.758 | 2.938 | 2.082 |
| CA759543 | LOC_Os03g63430 | -1.247 | -1.187 | 1.235 | 1.114 | 1.870 |
| CA759562 | LOC_Os12g21798 | 1.119 | 1.300 | 1.298 | 2.011 | 2.592 |
| CA759740 | **unknown** | 1.268 | 2.364 | 1.411 | 3.220 | 2.998 |
| CA760006 | LOC_Os02g43470 | -1.113 | 1.354 | 1.858 | 2.551 | 2.447 |
| CA760007 | LOC_Os01g48960 | 1.155 | 1.149 | 1.526 | 2.083 | 3.473 |
| CA760100 | **unknown** | -1.092 | 1.593 | 1.288 | 2.459 | 2.161 |
| CA760250 | LOC_Os01g56590 | -1.696 | -1.785 | 1.117 | 2.896 | 1.389 |
| CA760356 | LOC_Os06g09120 | -1.135 | -1.302 | 1.411 | 3.364 | 1.639 |
| CA761959 | LOC_Os06g12310 | 1.393 | 2.113 | 1.467 | 2.066 | 2.483 |
| CA761977 | LOC_Os11g08300 | -1.300 | -1.106 | 1.191 | 1.451 | 2.329 |
| CA762747 | LOC_Os02g35950 | -1.115 | -1.184 | -1.113 | 1.672 | 1.870 |
| CA762909 | LOC_Os01g59790 | -1.019 | -1.015 | 1.557 | 1.712 | 2.365 |
| CA763100 | LOC_Os10g30370 | 1.081 | -1.048 | -1.077 | 1.337 | 1.860 |
| CA763167 | LOC_Os05g15370 | 1.166 | 1.338 | 1.190 | 3.202 | 1.845 |
| CA763224 | LOC_Os06g04930 | -1.004 | -1.052 | -1.022 | 1.263 | 2.546 |
| CA763291 | LOC_Os03g16800 | 1.198 | 1.041 | 1.222 | 1.387 | 1.867 |
| CA763335 | LOC_Os12g19350 | 1.376 | 2.028 | 1.963 | 2.770 | 2.556 |
| CA763996 | LOC_Os02g57750 | -1.197 | 1.046 | 1.506 | 1.993 | 1.395 |
| CA764459 | LOC_Os09g36220 | -1.496 | -1.078 | 1.887 | 1.677 | 1.668 |
| CA764642 | LOC_Os01g22490 | -1.543 | 1.078 | -1.067 | 1.878 | 1.227 |
| CA764838 | LOC_Os10g34370 | -1.189 | 1.464 | 1.469 | 1.830 | 1.469 |
| CA764973 | LOC_Os01g27150 | -1.218 | 1.180 | 1.435 | 2.118 | 1.541 |
| CA765451 | LOC_Os05g35440 | 1.297 | 1.629 | 1.336 | 1.761 | 1.939 |
| CA765471 | LOC_Os07g25890 | -1.001 | 1.405 | 1.278 | 1.852 | 1.996 |
| CA765797 | LOC_Os02g42540 | -1.022 | 1.952 | 1.653 | 1.935 | 1.896 |
| CA766013 | LOC_Os05g34540 | 1.075 | 1.816 | 1.590 | 1.971 | 2.195 |
| CA766064 | LOC_Os02g01070 | 1.167 | 1.644 | 1.635 | 1.630 | 1.960 |
| CA766136 | LOC_Os01g61990 | -1.258 | 1.144 | 2.377 | 3.538 | 1.477 |
| CA766210 | LOC_Os01g73960 | -1.324 | -1.410 | 1.524 | 2.139 | 1.739 |
| CA766427 | LOC_Os04g30490 | -1.391 | -1.303 | 1.098 | 2.111 | 2.253 |
| CA767055 | LOC_Os05g42424 | -1.498 | 1.094 | 1.672 | 4.392 | 1.690 |
| CA767313 | LOC_Os06g49480 | 1.032 | -1.061 | 1.005 | 1.644 | 1.853 |
| CA767317 | LOC_Os03g32580 | 1.100 | -1.229 | 1.083 | 1.149 | 2.338 |
| CA997837 | LOC_Os08g13690 | -1.203 | 1.199 | 1.428 | 2.053 | 1.644 |

| **EST Acc.** | **Locus No.** | **0.5 hr** | **2 hr** | **6 hr** | **12 hr** | **24 hr** |
| --- | --- | --- | --- | --- | --- | --- |
| CA997842 | LOC_Os09g17830 | -1.451 | 1.269 | 1.631 | 3.023 | 1.763 |
| CA997843 | LOC_Os10g33800 | -1.028 | 1.344 | 1.178 | 2.402 | 2.113 |
| CA997862 | LOC_Os01g43370 | -1.436 | -1.166 | 1.556 | 2.092 | 1.449 |
| CA997866 | LOC_Os03g08050 | -1.505 | -1.029 | 1.351 | 2.104 | 1.519 |
| CA997870 | LOC_Os02g38920 | -1.352 | -1.139 | 1.070 | 2.283 | 1.735 |
| CA997913 | LOC_Os04g56520 | 1.389 | 1.370 | 1.558 | 2.486 | 1.913 |
| CA997943 | LOC_Os08g03290 | 1.050 | -1.005 | 1.626 | 2.546 | 2.844 |
| CA997954 | LOC_Os06g51220 | -1.505 | 1.084 | 1.354 | 2.021 | 2.405 |
| CA997955 | LOC_Os05g37970 | 1.559 | 2.094 | 1.883 | 1.706 | 1.471 |
| CA997967 | LOC_Os12g07140 | -1.338 | 1.746 | 1.575 | 1.896 | 1.430 |
| CA997973 | LOC_Os03g05980 | -1.149 | 1.279 | 1.344 | 1.876 | 1.482 |
| CA997981 | LOC_Os02g41630 | -1.249 | 1.342 | 1.119 | 2.048 | -1.112 |
| CA998007 | LOC_Os05g04510 | 1.185 | 2.021 | 1.578 | 1.353 | 1.506 |
| CA998008 | LOC_Os01g46070 | 1.218 | 1.810 | 1.781 | 2.249 | 2.041 |
| CA998014 | LOC_Os01g70310 | 1.180 | 1.774 | 1.278 | 2.435 | 1.384 |
| CA998024 | LOC_Os10g11260 | 1.123 | 1.908 | 1.978 | 2.826 | 1.800 |
| CA998038 | LOC_Os02g42320 | 1.067 | 1.534 | 2.390 | 1.453 | 1.076 |
| CA998043 | LOC_Os01g70940 | 1.023 | 1.734 | 1.813 | -1.041 | -1.455 |
| CA998055 | LOC_Os03g16690 | -1.175 | 1.348 | 1.966 | -1.061 | -1.251 |
| CA998060 | LOC_Os01g70170 | -1.149 | 1.117 | 1.717 | 1.501 | 2.014 |
| CA998073 | LOC_Os08g43090 | 1.017 | 1.849 | 2.235 | 1.136 | -1.246 |
| CA998085 | LOC_Os02g17390 | -1.604 | 1.176 | 2.065 | 2.065 | 1.721 |
| CA998086 | LOC_Os06g46770 | -1.740 | -1.111 | 1.456 | 2.095 | 1.022 |
| CA998134 | LOC_Os04g43800 | -1.101 | 1.231 | 2.195 | 1.099 | -1.381 |
| CA998153 | LOC_Os12g40890 | -1.144 | 1.382 | 1.783 | 2.870 | 1.318 |
| CA998160 | LOC_Os11g41130 | 1.374 | 2.036 | 1.571 | 1.170 | -1.032 |
| CA998167 | LOC_Os01g47550 | -1.282 | 1.107 | 1.840 | 2.408 | 1.284 |
| CA998323 | LOC_Os06g03850 | -1.375 | 1.826 | 1.788 | 2.233 | 1.496 |
| CA998346 | LOC_Os08g43540 | -1.311 | 1.607 | 1.821 | 2.048 | 1.339 |
| CA998351 | LOC_Os03g14650 | -1.197 | 1.507 | 1.764 | 2.579 | 1.344 |
| CA998362 | LOC_Os02g36740 | -1.216 | 1.430 | 1.691 | 1.632 | 2.420 |
| CA998424 | LOC_Os03g27820 | -1.017 | 1.924 | 1.842 | 2.291 | 1.576 |
| CA998447 | LOC_Os02g07260 | -1.229 | 1.312 | 1.400 | 2.554 | 1.584 |
| CA998461 | LOC_Os06g26234 | -1.685 | -1.166 | 1.227 | 1.933 | 1.066 |
| CA998474 | LOC_Os11g24070 | 1.010 | -1.189 | 1.133 | 1.935 | -1.138 |
| CA998493 | LOC_Os04g16780 | -1.467 | -1.146 | 1.466 | 2.024 | 1.176 |
| CA998788 | LOC_Os03g04000 | -1.057 | 1.229 | 1.865 | 1.081 | -1.284 |
| CA998959 | LOC_Os02g10070 | -1.337 | 1.105 | 1.432 | 1.857 | 1.243 |
| CA998999 | LOC_Os02g11050 | -1.030 | 1.472 | 1.761 | 1.301 | 1.968 |
| CA999019 | LOC_Os02g43930 | -1.516 | -1.031 | 1.223 | 1.646 | 2.238 |
| **EST Acc.** | **Locus No.** | **0.5 hr** | **2 hr** | **6 hr** | **12 hr** | **24 hr** |
| CA999155 | LOC_Os01g14690 | 1.171 | 1.655 | 1.751 | 1.923 | 1.250 |
| CA999193 | LOC_Os02g04800 | 1.188 | 1.843 | -1.021 | 1.041 | -1.258 |
| CA999235 | LOC_Os08g36900 | -1.255 | 1.845 | 1.722 | 2.270 | 1.551 |
| CA999284 | LOC_Os05g14180 | -1.279 | 1.038 | 1.861 | 1.847 | -1.054 |
| CA999320 | LOC_Os01g74650 | 1.221 | 1.291 | 1.805 | 1.725 | -1.029 |
| CA999596 | LOC_Os06g09890 | -1.174 | 1.080 | 1.575 | 2.471 | 1.512 |
| CA999612 | LOC_Os11g38810 | -1.363 | 1.132 | 1.724 | 2.678 | 1.761 |
| CA999633 | LOC_Os03g10340 | -1.242 | 1.340 | 1.361 | 1.647 | 2.058 |
| CA999664 | LOC_Os01g09560 | 1.179 | 1.870 | 1.710 | 2.210 | 2.045 |
| CA999670 | LOC_Os07g42370 | -1.308 | -1.280 | 1.523 | 1.819 | -1.008 |
| CA999789 | LOC_Os11g01530 | -1.457 | 1.417 | 1.449 | 2.844 | 3.297 |
| CA999799 | LOC_Os04g58110 | -1.464 | 1.103 | 1.714 | 2.846 | 1.441 |
| CA999846 | LOC_Os01g60190 | -1.175 | 1.256 | 1.181 | 2.123 | 1.666 |
| CA999922 | LOC_Os04g58850 | 1.175 | 1.953 | 1.349 | 1.282 | -1.064 |
| CA999930 | LOC_Os03g49600 | 1.060 | 1.690 | 1.622 | 2.175 | 1.911 |
| CA999946 | LOC_Os08g44450 | -1.524 | -1.143 | 1.420 | 2.031 | 1.437 |
| CA999999 | LOC_Os10g08550 | -1.649 | -1.113 | 1.465 | 2.253 | 1.430 |
| CA999999 | LOC_Os10g08550 | -1.649 | -1.113 | 1.465 | 2.253 | 1.430 |
| CB000008 | LOC_Os03g32470 | 1.069 | 1.675 | 1.267 | 2.130 | 1.740 |
| CB000017 | LOC_Os02g55890 | -1.018 | 1.507 | 1.270 | 2.053 | 1.455 |
| CB000020 | LOC_Os02g01560 | -1.276 | 1.246 | 1.090 | 1.854 | 1.688 |
| CB000117 | LOC_Os01g13030 | -1.051 | 1.565 | 1.893 | 2.747 | 1.323 |
| CB000149 | LOC_Os05g07700 | -2.068 | -1.235 | 1.809 | 1.689 | 1.133 |
| CB000189 | LOC_Os05g06430 | 1.178 | 1.520 | 1.501 | 2.422 | 2.175 |
| CB000247 | LOC_Os02g32814 | -1.257 | 1.053 | 1.389 | 1.948 | -1.071 |
| CB000323 | LOC_Os10g41520 | 1.143 | 1.997 | 1.420 | 1.373 | 1.592 |
| CB000459 | LOC_Os11g09280 | -1.256 | 1.194 | 1.067 | 1.912 | 1.837 |
| CB000478 | LOC_Os03g53230 | -1.730 | -1.668 | 2.427 | 2.572 | 1.751 |
| CB000495 | LOC_Os11g13940 | -1.435 | -1.119 | 1.657 | 3.002 | 1.476 |
| CB000523 | LOC_Os03g54890 | -1.762 | 1.123 | 1.271 | 1.816 | 1.379 |
| CB000626 | LOC_Os12g02320 | -1.197 | 1.026 | 1.977 | 4.601 | 1.630 |
| CB000645 | LOC_Os07g14850 | -1.226 | 1.569 | 1.291 | 2.147 | 1.343 |
| CB000724 | LOC_Os04g43680 | -1.464 | 1.103 | 1.714 | 2.846 | 1.441 |
| CB000751 | LOC_Os07g10720 | -1.346 | 1.256 | 1.398 | 2.098 | 1.143 |
| CB001067 | LOC_Os07g26110 | -1.344 | 1.170 | 1.485 | 1.826 | 1.394 |
| CB096284 | LOC_Os01g16030 | -1.149 | 1.351 | 1.451 | 2.328 | 1.891 |
| CB096560 | LOC_Os05g23860 | -1.078 | 1.112 | 1.608 | 2.228 | 1.458 |
| CB096630 | LOC_Os07g48100 | -1.489 | -1.544 | 1.117 | 2.533 | 2.439 |

| **EST Acc.** | **Locus No.** | **0.5 hr** | **2 hr** | **6 hr** | **12 hr** | **24 hr** |
| --- | --- | --- | --- | --- | --- | --- |
| CB096828 | LOC_Os02g54160 | -1.216 | -1.256 | 1.386 | 1.924 | 2.238 |
| CB096933 | LOC_Os05g11780 | -1.094 | 1.575 | 1.638 | 2.081 | 1.117 |
| CB097201 | LOC_Os05g50550 | -1.336 | 1.241 | 1.644 | 2.373 | 1.543 |
| EL586673 | **unknown** | -1.300 | 1.101 | 1.854 | 1.748 | 1.618 |
| EL586674 | **unknown** | -1.248 | 1.341 | 1.472 | 1.978 | 1.289 |
